# Author Correction: Prediction-based highly sensitive CRISPR off-target validation using target-specific DNA enrichment

**DOI:** 10.1038/s41467-020-20559-5

**Published:** 2021-01-04

**Authors:** Seung-Hun Kang, Wi-jae Lee, Ju-Hyun An, Jong-Hee Lee, Young-Hyun Kim, Hanseop Kim, Yeounsun Oh, Young-Ho Park, Yeung Bae Jin, Bong-Hyun Jun, Junho K. Hur, Sun-Uk Kim, Seung Hwan Lee

**Affiliations:** 1 Department of Medicine, Graduate School, Kyung Hee University, Seoul, Republic of Korea; 2 Futuristic Animal Resource & Research Center (FARRC), Korea Research Institute of Bioscience and Biotechnology (KRIBB), Cheongju, Republic of Korea; 3 Department of Bioscience and Biotechnology, Konkuk University, Seoul, 143-701 Korea; 4 Department of Functional Genomics, KRIBB School of Bioscience, Korea University of Science and Technology (UST), Daejeon, Korea; 5 National Primate Research Center (NPRC), Korea Research Institute of Bioscience and Biotechnology (KRIBB), Cheongju, Korea; 6 School of Life Sciences and Biotechnology, BK21 Plus KNU Creative BioResearch Group, Kyungpook National University, Daegu, Republic of Korea; 7 Division of Biotechnology, College of Life Science and Biotechnology, Korea University, Seoul, 02841 Republic of Korea; 8 Department of Pathology, College of Medicine, Kyung Hee University, Seoul, Republic of Korea; 9 Department of Biomedical Science, Graduate School, Kyung Hee University, Seoul, Republic of Korea; 10 Department of Medical Genetics, College of Medicine, Hanyang University, Seoul, Republic of Korea

Correction to: *Nature Communications* 10.1038/s41467-020-17418-8, published online 17 July 2020.

In the original version of this Article, the Acknowledgements incorrectly stated a grant from the National Research Foundation funded by the Korean Ministry of Education, Science and Technology as “NRF-2019M3A9H110378”. It should be stated as “NRF-2019M3A9H1103783”. This has been corrected in the PDF and HMTL version of the article.

