# Supplementary material for: Prediction-based highly sensitive CRISPR off-target validation using target-specific DNA enrichment
Source: Nat Commun. 2020 Jul 17;11:3596. doi: 10.1038/s41467-020-17418-8 (PMC7368065; doi:10.1038/s41467-020-17418-8)
Supplement: Supplementary file 1 — Supplementary Information [file 41467_2020_17418_MOESM1_ESM.pdf]

Supplementary Tables

Supplementary Table 1. Sequence information for crRNAs used in this study. Direct repeat sequences of the AsCas12a and SpCas9 in guide RNAs are shown in blue.

target	crRNA Sequence		
AGBL1 on	5`	GAAUUUCUACUCUUGUAGAUGAUUGAAGGAAAGUUACAAAGG	3`
RPL32P3 on	5`	GAAUUUCUACUCUUGUAGAUGGGUGAUCAGACCCAACAGCAGG	3`
RPL32P3 off1	5`	GAAUUUCUACUCUUGUAGAUGGGUGAUCAGACCCAACACCAGG	3`
RPL32P3 off2	5`	GAAUUUCUACUCUUGUAGAUGGGUGAUCAGACCCAACACCAGG	3`
RPL32P3 off3	5`	GAAUUUCUACUCUUGUAGAUGGGUGAUCAGACCCAACACCAGG	3`
RPL32P3 off4	5`	GAAUUUCUACUCUUGUAGAUGGGUGAUCAGGCCCAACACCAGG	3`
RPL32P3 off5	5`	GAAUUUCUACUCUUGUAGAUGGGUGAUCAGACCCAACCCCAGG	3`
RPL32P3 off6	5`	GAAUUUCUACUCUUGUAGAUGGGUGAUCAGACCUAACACUAGG	3`
RPL32P3 off7	5`	GAAUUUCUACUCUUGUAGAUGGGUGAUCCAACCCAACACCAGG	3`
RPL32P3 off8	5`	GAAUUUCUACUCUUGUAGAUGGGUGGCCAGACCCAACACCAGG	3`
RPL32P3 off9	5`	GAAUUUCUACUCUUGUAGAUGGGUGGACAGACCCAACACCAGG	3`
RPL32P3 off10	5`	GAAUUUCUACUCUUGUAGAUGGGUGUUCAGGACCAACAACAGG	3`
PSMB2 on	5`	GAAUUUCUACUCUUGUAGAUCUCUGAGUGUACAAAAGAUGGUG	3`
PSMB2 off2	5`	GAAUUUCUACUCUUGUAGAUCAAGUAUAUACCCAGUGCUGAGC	3`
PSMB2 off3	5`	GAAUUUCUACUCUUGUAGAUCAGGUCACUAAAAAAUUAAAUGA	3`
PSMB2 off4	5`	GAAUUUCUACUCUUGUAGAUCAUGUUUACACAUUAUUUAACUC	3`
FAT3 on	5`	GAAUUUCUACUCUUGUAGAUAAGGGAAGAAACCCUGAAACUCU	3`
FAT3 off3	5`	GAAUUUCUACUCUUGUAGAUAAGGGGACAGCCACUACUGAGGC	3`
DNMT1 site3 on	5`	GAAUUUCUACUCUUGUAGAUCUGAUGGUCCAUGUCUGUUACUC	3`
DNMT1 site3 off1	5`	GAAUUUCUACUCUUGUAGAUCUGAUGGUCCACGCCUGUUAACA	3`
DNMT1 site3 off2	5`	GAAUUUCUACUCUUGUAGAUCUGAUGGUCCAUACCUGUUAACA	3`
DNMT1 site3 off3	5`	GAAUUUCUACUCUUGUAGAUCUGAUGGUCCAUGUCUGAAUUAG	3`
HEK site4 on	5	GGCACUGCGGCUGGAGGUGGGUUUUAG AGC UAG AAA UAG CAA G UUAAA AUAAG GCUAG UCCGU UAUCA ACUUG AAAAA GUGGC ACCGA GUCGG UGC	3
HEK site4 off1	5	UGCACUGCGGCCGGAGGAGGGUUUUAG AGC UAG AAA UAG CAA G UUAAA AUAAG GCUAG UCCGU UAUCA ACUUG AAAAA GUGGC ACCGA GUCGG UGC	3
HEK site4 off2	5	GGCAUCACGGCUGGAGGUGGGUUUUAG AGC UAG AAA UAG CAA G UUAAA AUAAG GCUAG UCCGU UAUCA ACUUG AAAAA GUGGC ACCGA GUCGG UGC	3

HEK site4 off3	5	AGCAGUGCGGCUAGAGGUGGGUUUUAG AGC UAG AAA UAG CAA G UUAAA AUAAG GCUAG UCCGU UAUCA ACUUG AAAAA GUGGC ACCGA GUCGG UGC	3
HEK site4 off4	5	GGCACUGAGAAAGGAGGUGGGUUUUAG AGC UAG AAA UAG CAA G UUAAA AUAAG GCUAG UCCGU UAUCA ACUUG AAAAA GUGGC ACCGA GUCGG UGC	3
HEK site4 off5	5	GGGCAUGCGGCUGGAAGUGGGUUUUAG AGC UAG AAA UAG CAA G UUAAA AUAAG GCUAG UCCGU UAUCA ACUUG AAAAA GUGGC ACCGA GUCGG UGC	3
HEK site4 off6	5	GGCACAGGGGCUGGAGGUGGGUUUUAG AGC UAG AAA UAG CAA G UUAAA AUAAG GCUAG UCCGU UAUCA ACUUG AAAAA GUGGC ACCGA GUCGG UGC	3
RNF2 on	5	GUCAUCUUAGUCAUUACCUGGUUUUAG AGC UAG AAA UAG CAA G UUAAA AUAAG GCUAG UCCGU UAUCA ACUUG AAAAA GUGGC ACCGA GUCGG UGC	3
RNF2 off1	5	GGUAUCUAAGUCAUUACCUGGUUUUAG AGC UAG AAA UAG CAA G UUAAA AUAAG GCUAG UCCGU UAUCA ACUUG AAAAA GUGGC ACCGA GUCGG UGC	3
RNF2 off2	5	GUAAUCUGAGUCAUUUCCUGGUUUUAG AGC UAG AAA UAG CAA G UUAAA AUAAG GCUAG UCCGU UAUCA ACUUG AAAAA GUGGC ACCGA GUCGG UGC	3
RNF2 off3	5	GUCAUCCUAGUGCUUACCUGGUUUUAG AGC UAG AAA UAG CAA G UUAAA AUAAG GCUAG UCCGU UAUCA ACUUG AAAAA GUGGC ACCGA GUCGG UGC	3
ZNF609 (Spacer 8)	5	CCCCCACCAAAGCCCATGTAGUUUUAG AGC UAG AAA UAG CAA G UUAAA AUAAG GCUAG UCCGU UAUCA ACUUG AAAAA GUGGC ACCGA GUCGG UGC	3
RP11-77I22.4 (Spacer 15A)	5	GGCAGTGCAGATGAAAAACTGUUUUAG AGC UAG AAA UAG CAA G UUAAA AUAAG GCUAG UCCGU UAUCA ACUUG AAAAA GUGGC ACCGA GUCGG UGC	3

Supplementary Table 2. Sequence information for DNA targets amplified in this study. PAM sequences (TTTN for AsCas12a and NGG for SpCas9 effector) are shown in blue color and mismatched sequence to each on-target sites are shown in red color respectively.

Target name	Mismatch	Sequence	Position		Direction
AGBL1 on	-	**TTTA** GATTGAAGGAAAAGTTACAAAGG	chr15	86124495	-
RPL32P3 on	-	**TTTG** GGGTGATCAGACCCAACAGCAGG	chr3	129389649	-
RPL32P3 off1	1	**TTTG** GGGTGATCAGACCCAACAcCAGG	chr8	108739813	+
RPL32P3 off2	1	**TTTG** GGGTGATCAGACCCAACAcCAGG	chr2	88944272	-
RPL32P3 off3	1	**TTTG** GGGTGATCAGACCCAACAcCAGG	chr6	33355560	-
RPL32P3 off4	2	**TTTG** GGGTGATCAGgCCCAACAcCAGG	chr1	205858150	-
RPL32P3 off5	2	**TTTG** GGGTGATCAGACCCAACccCAGG	chr15	78621826	+
RPL32P3 off6	3	**TTTG** GGGTGATCAGACCtAACActAGG	chr3	109693289	-
RPL32P3 off7	3	**TTTG** GGGTGATCcaACCCAACAcCAGG	chr4	43472802	-
RPL32P3 off8	3	**TTTG** GGGTGgcCAGACCCAACAcCAGG	chr19	53480825	+
RPL32P3 off9	3	**TTTG** GGGTGgaCAGACCCAACAcCAGG	chr20	42901255	-
RPL32P3 off10	4	**TTTT** GGGTGtTCAGgaCCAACAaCAGG	chr12	133092583	+

PSMB2 on	-	GTAAACAAAGCATAGACTGA **GGG**	chr1	35631454	+
PSMB2 off2	3	GcAAACAAAaCAgAGACTGA **AGG**	chr3	77072677	-
PSMB2 off3	3	GTAAACAAcaCAgAGACTGA **AGG**	chr7	112493828	-
PSMB2 off4	3	GgAAACAgAGCATAGAaTGA **TGG**	chr1	208960762	+
FAT3 on	-	GAGCTGCTTAAGCATTTCAA **GGG**	chr11	92532744	-
FAT3 off3	3	GAGaTGCagAAGCATTTCAA **GGG**	chr18	25854400	+
DNMT1 site3 on	-	**TTTC** CTGATGGTCCATGTCTGTTACTC	chr19	10,133,766	-
DNMT1 site3 off1	5	**TTTC** CTGATGGTCCAcGcCTGTTAaca	chrX	98291178	-
DNMT1 site3 off2	5	**TTTC** CTGATGGTCCATacCTGTTAaca	chrX	93421364	-
DNMT1 site3 off3	6	**TTTC** CTGATGGTCCATGTCTGaattag	chr1	213204013	+
HEK site4 on	-	GGCACTGCGGCTGGAGGTGG **GGG**	chr20	32761949	+
HEK site4 off1	3	tGCACTGCGGCcGGAGGaGG **TGG**	chr20	61435489	+
HEK site4 off2	3	GGCAtcaCGGCTGGAGGTGG **AGG**	chr10	75343344	+
HEK site4 off3	3	aGCAgTGCGGCTaGAGGTGG **TGG**	chr13	38688774	+
HEK site4 off4	4	GGCACTGaGaaaGGAGGTGG **AGG**	chr12	55034151	+
HEK site4 off5	4	GGgcaTGCGGCTGGAaGTGG **TGG**	chr22	41224051	+
HEK site4 off6	2	GGCACaGgGGCTGGAGGTGG **GGC**	chr3	195823597	-
RNF2 on	-	GTCATCTTAGTCATTACCTG **AGG**	chr1	185087634	-
RNF2 off1	3	GgtATCTaAGTCATTACCTG **TGG**	chr5	92701252	-
RNF2 off2	3	GTaATCTgAGTCATTtCCTG **GGG**	chr10	129047186	+
RNF2 off3	3	GTCATCcTAGTgcTTACCTG **AGG**	chr8	755959	+
ZNF609 (Spacer 8A)	-	CCCCCACCAAAGCCCATGTA **AGG**	chr15	64593305	+
RP11-77I22.4 (Spacer 15A)	-	GGCAGTGCAGATGAAAAACT **GGG**	chr12	30862020	-

Supplementary Table 3. Sequence information for DNA primers used in this study. Primer sequence information to amplify the target gene was indicated. The base sequences of the forward and reverse adapter primers used in the next generation sequencing are shown in green and blue, respectively.

Target gene (primer direction)	DNA sequence (5' to 3')
AGBL1 on-target F1	TGCCCTGCCATTATATAGACTGTT
AGBL1 on-target R1	AAAATACAAAATGTAGCGGGGCA
AGBL1 on-target F2	CATTACAACCTTCTTCTGTTTGTC
AGBL1 on-target R2	ACCAGAGTGAGACTCTGTCT
FAT3 on-target F1	AGGAGTTTGAAGCTGTAGTAAG
FAT3 on-target F2	CGGGAGACTCTGTCTCTTAA
FAT3 on-target R1	AACCCGCCTTTTGTTACAAGTT
FAT3 on-target R2	CAAAACTTCAAACAGAATACATGTG
FAT3 off-target3 F1	GAGAATACTGTGCAGCCAGAG

FAT3 off-target3 F2	AGAAGTTGAGAGACCCTCAGAA
FAT3 off-target3 R1	CCAGCACACCTTGGTAATCTG
FAT3 off-target3 R2	GTAACCTCTCCTAACCAGCACA
RPL32P3 on-target F1	ACCATGTCGTCCTGTATGATC
RPL32P3 on-target F2	CTTCCTGAAGACACCGATTCT
RPL32P3 on-target R1	AGAGACCAACAATGACCTGTTT
RPL32P3 on-target R2	AGAACCTTGCACATGAAATGTG
RPL32P3 off-target1 F1	CAGCAATTGCTATCTGTTGTAGTT
RPL32P3 off-target1 F2	TCTGTTGTAGTTCTTGTGGGTT
RPL32P3 off-target1 R1	AGTGTTGGGAAGAAAGCTGAG
RPL32P3 off-target1 R2	TCCTCTGGAATGTGTCTAGACT
RPL32P3 off-target2 F1	AGTCTCCATTCTGTTCATGCC
RPL32P3 off-target2 F2	TTCATGCCCACAATGGTGATAT
RPL32P3 off-target2 R1	CTCTCCAAGGATCGATTGTATCT
RPL32P3 off-target2 R2	CTGCTCTCCATTATCTCAAGTA
RPL32P3 off-target3 F1	GTAAGGGTGCACCCTTCTATA
RPL32P3 off-target3 F2	GTACCTAGCCTTGCTGAGAA
RPL32P3 off-target3 R1	CCTTTGGAATGTGTCCAGAC
RPL32P3 off-target3 R2	TGCTGGCTTCTTGCTTCTAG
RPL32P3 off-target4 F1	GCAAAACTGCTCCACTGTACT
RPL32P3 off-target4 F2	ACCTTGGTTCTTCAGAGTGC
RPL32P3 off-target4 R1	TGTTGGTTCCTTGCTTCTTACTT
RPL32P3 off-target4 R2	CCTCCATTATCTCAAGCAGCA
RPL32P3 off-target5 F1	GTGACAGAGTGAGACTTCATCT
RPL32P3 off-target5 F2	GGTGGGTGGAAGTTAGTTGA
RPL32P3 off-target5 R1	ACATTCTTGGGGTTGCAGATG
RPL32P3 off-target5 R2	CTCTCAGATTGCTCCATCAGAA
RPL32P3 off-target6 F1	GTAGAGAACTGAAGAAAGATCTGC
RPL32P3 off-target6 F2	AGAGCAGAGGTCTCCATTCTTA
RPL32P3 off-target6 R1	CACTATTATTGCGTCTAAGTCCTT
RPL32P3 off-target6 R2	CTGGGGTCTAAGGTCTAAAAC
RPL32P3 off-target7 F1	CTATGGGATACAGCCAAAACAGT
RPL32P3 off-target7 F2	ACAGCCAAAACAGTACTAAGAG
RPL32P3 off-target7 R1	TGATAGCTGTGGTGGTTTTACAA
RPL32P3 off-target7 R2	AGCTGAGTGTTGGGAGAGAA
RPL32P3 off-target8 F1	TCAGTGCCTACCTGGTAAAGAT
RPL32P3 off-target8 F2	GTCATCTGTGGACATCTTGAG
RPL32P3 off-target8 R1	TGATCTCAAGTCGCAGAACAT
RPL32P3 off-target8 R2	AAGTCGCAGAACATGTTCCATA
RPL32P3 off-target9 F1	GTGGACATACACAAGTGTTTCA
RPL32P3 off-target9 F2	GTATGACATTCTGTGATGGGAG
RPL32P3 off-target9 R1	CCAGGACTGATTGTATCTTGAGT
RPL32P3 off-target9 R2	CACTATCTCAAGCAGCAGAACA
RPL32P3 off-target10 F1	CTAACAGAATACGATGCTGCC
RPL32P3 off-target10 F2	TACGATGCTGCCAATCAAAAGT
RPL32P3 off-target10 R1	GCTGATACTGCAGTGGTACT
RPL32P3 off-target10 R2	TTCACAGGCTCCATCTCTCT

PSMB2 on-target F1	CTTCATATTGGTGTGTCCCAAC
PSMB2 on-target F2	AATGAACAAGTAGCAACAGGAGG
PSMB2 on-target R1	AGCTGGGATTACAGGCATGTAC
PSMB2 on-target R2	TCCCAAAGCTCTAGGATTACAG
PSMB2 off-target2 F1	GCCTAGCAGATTTATTTTCTGTTC
PSMB2 off-target2 F2	TTCTGACTTCTGCTACTCATTGC
PSMB2 off-target2 R1	ACAAACTCAAGACTGTCACTGATT
PSMB2 off-target2 R2	GGTGGGGAACTTGTGATCTAG
PSMB2 off-target3 F1	ACCCGAGCAGCACTACTTTTC
PSMB2 off-target3 F2	TGCTTGCTCAAACCTGCTATC
PSMB2 off-target3 R1	CTACAAAGGGTGTCAGAGGCA
PSMB2 off-target3 R2	GGTGGGACTAGGAATTCCTG
PSMB2 off-target4 F1	CCAAAGGAAGATACAGCAGTGT
PSMB2 off-target4 F2	GTAGTCAACCTCAGTGTCCAT
PSMB2 off-target4 R1	AATGCATTTCTGGTTACCCTGTT
PSMB2 off-target4 R2	GTGTGAAGTGGTCAACTACAAG
DNMT1-site3 on-target F1	CAAAGCCATTGGCTTGGAGAT
DNMT1-site3 on-target F2	AGATCAAGCTTTGTATGTTGGCC
DNMT1-site3 on-target R1	AGAAGTCCCGTGCAAATCAC
DNMT1-site3 on-target R2	GCAAATCACGAATACCCACCC
DNMT1-site3 off-target1 F1	TGTTGCAAGTCCCATGAGGA
DNMT1-site3 off-target1 F2	CAGGGAACTCTAATCTCACAAT
DNMT1-site3 off-target1 R1	GAACAGGAAAGAAAGGAAAATGAG
DNMT1-site3 off-target1 R2	CCTCTTTCCCATGATTCTTCC
DNMT1-site3 off-target2 F1	AGCAGGTCATTGGCAATGATAC
DNMT1-site3 off-target2 F2	CAAATGTTTGTGCAGGTTGATGTT
DNMT1-site3 off-target2 R1	GGTTTAGAGCAGGAGTGAAAGT
DNMT1-site3 off-target2 R2	AAGAAAGGAAAGTTCACTTGGAAG
DNMT1-site3 off-target3 F1	AACCCTGCTACCTACTGAGAAT
DNMT1-site3 off-target3 F2	CATTAGCCTGTGTTTTCACATAAG
DNMT1-site3 off-target3 R1	TTAATAGCATCAAAGGCAAACCAT
DNMT1-site3 off-target3 R2	ATGATGGGAAAGTGTGCAAATAG
HEK site4 on-target F1	GCTTACAGGCGATATAAATCATTC
HEK site4 on-target F2	CCACAAGCAGGTAAACAAGCA
HEK site4 on-target R1	TGGGGGATCAGAAGCCCTAA
HEK site4 on-target R2	GACGTCCAAAACCAGACTCC
HEK site4 off-target1 F1	GTCCTGCAGCCTTCATTCCT
HEK site4 off-target1 F2	GGATTGTGAGATTGTGTAGGCA
HEK site4 off-target1 R1	TGGAAGGTCACAGAACACATGT
HEK site4 off-target1 R2	ACATGAGGCAGCTGGTGTCT
HEK site4 off-target2 F1	GACAAACGGTCACTTAAATGCG
HEK site4 off-target2 F2	GAGACCCAAGGACTGGGTAA
HEK site4 off-target2 R1	GCAGCTTTTTCCCAACCTCT
HEK site4 off-target2 R2	CAAGTCTCTGCCCTAAAGAATC
HEK site4 off-target3 F1	ATGATGGAGGTGGACCAGTTT
HEK site4 off-target3 F2	TTCAACCTTACTTCTCCATTCCA
HEK site4 off-target3 R1	CTTCCTCCAAAGGCCTCTGA

HEK site4 off-target3 R2	AGAATCATCTGAATCCATGTCAGT
HEK site4 off-target4 F1	ATGTCAGCTGACATGTTTCTAATTT
HEK site4 off-target4 F2	CAGTACATGTTGAATACATACACAT
HEK site4 off-target4 R1	TTGGAGAGAAGAGGTTTCAGGA
HEK site4 off-target4 R2	AGCCTGGCCTATTGCTCCTA
HEK site4 off-target5 F1	CAGCTCTGGCACAAATGAGT
HEK site4 off-target5 F2	TTGGTGGCAAGAAGTGGCAT
HEK site4 off-target5 R1	TGGTAGCATCTGGGTTCAAATC
HEK site4 off-target5 R2	CCTGCTGTGACGAGTAGGAA
HEK site4 off-target6 F1	CTTCTGGAGCTGCCATCTAC
HEK site4 off-target6 F2	TCCAACCTCTACATTTGTTCAG
HEK site4 off-target6 R1	CAAAATGCTGGGATTACAGGCA
HEK site4 off-target6 R2	AAACATTCATAAGCCGTTATTGCC
RNF2 on-target F1	ATCCAGTGTTAAGCATGTTTGTTG
RNF2 on-target F2	CTGTTTTATTCACCACTGTTCAC
RNF2 on-target R1	TTATAGCTGCTTCTCTGTGTCA
RNF2 on-target R2	CAAAAGTTTCCATCAAGCCTCTT
RNF2 off-target1 F1	AATTTAGCCCACATCACTGGAG
RNF2 off-target1 F2	GGAGTGAGATGCCATCTTATCA
RNF2 off-target1 R1	AAAAGTCAACATCTGAAACGTGCT
RNF2 off-target1 R2	CTAGATGCTTACCTTTGTGACC
RNF2 off-target2 F1	TCCTCCTGGATACTGATATACTT
RNF2 off-target2 F2	TCTAATGTCCTGGGATGCTTCT
RNF2 off-target2 R1	ATAGGTGCACATGCTACGTTATTA
RNF2 off-target2 R2	TCCGGCTCCAACCAAGTTAA
RNF2 off-target3 F1	GGAGCTCTTTGTGAATCTGAG
RNF2 off-target3 F2	TTGAAAGAGCAAAGTGCTGGG
RNF2 off-target3 R1	TCCCACAACGACATCGTCTTT
RNF2 off-target3 R2	CTAAACAGCATGAGCCCATCA
ZNF609 (Spacer 8A) F1	GCAGGAGAATTGCTTGAATCTA
ZNF609 (Spacer 8A) F2	TATTCGCTGCACTAACTGTGC
ZNF609 (Spacer 8A) R1	TCACTTGAGTCTAGGAGTTTGA
ZNF609 (Spacer 8A) R2	GACCCTGTCTCAAAAACAAACAA
RP11-77I22.4 (Spacer 15A) F1	TAAGTGTTAAGCTGGAAGGCCA
RP11-77I22.4 (Spacer 15A) F2	AAACATGGAACCCAAAGGAATTG
RP11-77I22.4 (Spacer 15A) R1	TCACTGTTCTGGAGGCTGAGAA
RP11-77I22.4 (Spacer 15A) R2	TGGTGCCTTCTAACTGTGTTC
AGBL1_on_Adaptor_F	ACACTCTTTCCCTACACGACGCTCTTCCGATCT CTTAAGGAAACAGAAGAGAAATCTGCGTG
AGBL1_on_Adaptor_R	GTGACTGGAGTTCAGACGTGTGCTCTTCCGATCT ATTATAAGATGTTGACAATAACACAACAGG
FAT3_on_Adaptor_F	ACACTCTTTCCCTACACGACGCTCTTCCGATCT GTGGATTGTTTCATTCCAATGATGAAACAA
FAT3_on_Adaptor_R	GTGACTGGAGTTCAGACGTGTGCTCTTCCGATCT CATTTTGAACAGCCTTCTCCAGAAAACA
FAT3_off3_Adaptor_F	ACACTCTTTCCCTACACGACGCTCTTCCGATCT GGAAAATGAGCACTTAGCAATCAATTTG

FAT3_off3_Adaptor_R	GTGACTGGAGTTCAGACGTGTGCTCTTCCGATCT CTGTTTCTTTTTCTTGTTACTGCAGCA
RPL32P3_on_Adaptor_F	ACACTCTTTCCCTACACGACGCTCTTCCGATCT TGGGACAGGGTGGGTTACCTTG
RPL32P3_on_Adaptor_R	GTGACTGGAGTTCAGACGTGTGCTCTTCCGATCT ATCCTCACCACCTGTTTGTTGCA
RPL32P3_off1_Adaptor_F	ACACTCTTTCCCTACACGACGCTCTTCCGATCT TAGCTAGGGTGACTTGGCTAGCTTG
RPL32P3_off1_Adaptor_R	GTGACTGGAGTTCAGACGTGTGCTCTTCCGATCT GGTCCCACTTCTCTCTCTCAAATTGT
RPL32P3_off2_Adaptor_F	ACACTCTTTCCCTACACGACGCTCTTCCGATCT AAGCCCTGCAAATGTAAAAATCATAACA
RPL32P3_off2_Adaptor_R	GTGACTGGAGTTCAGACGTGTGCTCTTCCGATCT TGCCCACATTTCTCTCTCAAACTGTC
RPL32P3_off3_Adaptor_F	ACACTCTTTCCCTACACGACGCTCTTCCGATCT AAAAATCAAGACCTACCCAGTGCAAG
RPL32P3_off3_Adaptor_R	GTGACTGGAGTTCAGACGTGTGCTCTTCCGATCT CTTTGCAACCTCCATACTAGTATTGGC
RPL32P3_off4_Adaptor_F	ACACTCTTTCCCTACACGACGCTCTTCCGATCT AGCATTTCTTTACCATGGTCTTCATAGC
RPL32P3_off4_Adaptor_R	GTGACTGGAGTTCAGACGTGTGCTCTTCCGATCT TTCGCAGCCTCCAATCTAGTGTT
RPL32P3_off5_Adaptor_F	ACACTCTTTCCCTACACGACGCTCTTCCGATCT GTACCTGGAGGTTTGTTATACTGTTCTCT
RPL32P3_off5_Adaptor_R	GTGACTGGAGTTCAGACGTGTGCTCTTCCGATCT GCCTGGTCCCGCTTTCTCTCTT
RPL32P3_off6_Adaptor_F	ACACTCTTTCCCTACACGACGCTCTTCCGATCT ATGCATCCATGTATATTTTGGAGCATTCA
RPL32P3_off6_Adaptor_R	GTGACTGGAGTTCAGACGTGTGCTCTTCCGATCT TCTTTTTTGCAAAGACCCACCTTATGT
RPL32P3_off7_Adaptor_F	ACACTCTTTCCCTACACGACGCTCTTCCGATCT ACAAAACTGAACAAACCTTTAGCCAGA
RPL32P3_off7_Adaptor_R	GTGACTGGAGTTCAGACGTGTGCTCTTCCGATCT GCAGCCTCCATACTAGCATTGG
RPL32P3_off8_Adaptor_F	ACACTCTTTCCCTACACGACGCTCTTCCGATCT GCTATTAAGTGCATGTTTCCCTCAAGG
RPL32P3_off8_Adaptor_R	GTGACTGGAGTTCAGACGTGTGCTCTTCCGATCT CTGGACCCACCTTATGCATTCTTAACT
RPL32P3_off9_Adaptor_F	ACACTCTTTCCCTACACGACGCTCTTCCGATCT AGGCACTGTGCTGTCTTACATGTTATT
RPL32P3_off9_Adaptor_R	GTGACTGGAGTTCAGACGTGTGCTCTTCCGATCT CCTGGACACACTTTCTCTCTCAAACT
RPL32P3_off10_Adaptor_F	ACACTCTTTCCCTACACGACGCTCTTCCGATCT TATGGCTTGTGATTTCTTGTTTCCATAACT
RPL32P3_off10_Adaptor_R	GTGACTGGAGTTCAGACGTGTGCTCTTCCGATCT AGACCAGGTTTACAGAAGGCTAGAA
PSMB2_on_Adaptor_F	ACACTCTTTCCCTACACGACGCTCTTCCGATCT AGTAAAGCAGAAGGAATAACAGTGCCC

PSMB2_on_Adaptor_R	GTGACTGGAGTTCAGACGTGTGCTCTTCCGATCT<br>TTCTGTTTGTGGCCAAGAATTGCTGT
PSMB2_off2_Adaptor_F	ACACTCTTTCCCTACACGACGCTCTTCCGATCT<br>ACACAAACCAAGGGTGATGAAGTTT
PSMB2_off2_Adaptor_R	GTGACTGGAGTTCAGACGTGTGCTCTTCCGATCT<br>CATAATTTCGAAACATATACACAATGGG
PSMB2_off3_Adaptor_F	ACACTCTTTCCCTACACGACGCTCTTCCGATCT<br>GACAAGAGATTACTAGTGTTGCCTAAACA
PSMB2_off3_Adaptor_R	GTGACTGGAGTTCAGACGTGTGCTCTTCCGATCT<br>TCAGCATTTTCTTCTGTATCATGGGAG
PSMB2_off4_Adaptor_F	ACACTCTTTCCCTACACGACGCTCTTCCGATCT<br>GGAGGACATTATCTTAAATGAAACAACTC
PSMB2_off4_Adaptor_R	GTGACTGGAGTTCAGACGTGTGCTCTTCCGATCT<br>CCACATTGAACCCAACAAGCAACTT
DNMT1-site3_on_Adaptor_F	ACACTCTTTCCCTACACGACGCTCTTCCGATCT<br>GTTGCACGTGTCAAGTGCTTAGAG
DNMT1-site3_on_Adaptor_R	GTGACTGGAGTTCAGACGTGTGCTCTTCCGATCT<br>GACTGAACACTCCTCAAACGGTC
DNMT1-site3_off1_Adaptor_F	ACACTCTTTCCCTACACGACGCTCTTCCGATCT<br>CTACCCCCACCACTAGAAATGCCA
DNMT1-site3_off1_Adaptor_R	GTGACTGGAGTTCAGACGTGTGCTCTTCCGATCT<br>TTTGTCTCTTAACATGCATGCCTAGGAA
DNMT1-site3_off2_Adaptor_F	ACACTCTTTCCCTACACGACGCTCTTCCGATCT<br>CCTCAACCACTAAATATGTTATTTAGTGGT
DNMT1-site3_off2_Adaptor_R	GTGACTGGAGTTCAGACGTGTGCTCTTCCGATCT<br>CCTTTTCCGATGGAGTGTACTCAGAAGAT
DNMT1-site3_off3_Adaptor_F	ACACTCTTTCCCTACACGACGCTCTTCCGATCT<br>GGTCTCAAATAAGTTTGAGAATGAATGTG
DNMT1-site3_off3_Adaptor_R	GTGACTGGAGTTCAGACGTGTGCTCTTCCGATCT<br>AGAGCTGAAAGTTTAGCATGGAGG
HEK site4_on_Adaptor_F	ACACTCTTTCCCTACACGACGCTCTTCCGATCT<br>AGAGGGTCCAAAGCAGGATGACAG
HEK site4_on_Adaptor_R	GTGACTGGAGTTCAGACGTGTGCTCTTCCGATCT<br>CTTTCAACCCGAACGGAGACACACA
HEK site4_off1_Adaptor_F	ACACTCTTTCCCTACACGACGCTCTTCCGATCT<br>TTCTGAGACTCATAGCTGGGGCTGAA
HEK site4_off1_Adaptor_R	GTGACTGGAGTTCAGACGTGTGCTCTTCCGATCT<br>CCTCGGAGTCCTCAAGTATCACTGTCC
HEK site4_off2_Adaptor_F	ACACTCTTTCCCTACACGACGCTCTTCCGATCT<br>TCATTTCCACCAGAACTCAGCCCAG
HEK site4_off2_Adaptor_R	GTGACTGGAGTTCAGACGTGTGCTCTTCCGATCT<br>TGTTTCCACCCTCGGTTCCTCCACAA
HEK site4_off3_Adaptor_F	ACACTCTTTCCCTACACGACGCTCTTCCGATCT<br>CGGTCTTATTCTCTATGAGGGTCAGTCTC
HEK site4_off3_Adaptor_R	GTGACTGGAGTTCAGACGTGTGCTCTTCCGATCT<br>CAGCTCAGCCACTGTAAAGCTCTT
HEK site4_off4_Adaptor_F	ACACTCTTTCCCTACACGACGCTCTTCCGATCT<br>GCAACTCTACAGGCTGAGTTCTTTCTT

HEK site4_off4_Adaptor_R	GTGACTGGAGTTCAGACGTGTGCTCTTCCGATCT TTAGATCAAGGAAGAACGTTTTCCATTACC
HEK site4_off5_Adaptor_F	ACACTCTTTCCCTACACGACGCTCTTCCGATCT CCTGTGTTCTGACGTCGTTTCAGATG
HEK site4_off5_Adaptor_R	GTGACTGGAGTTCAGACGTGTGCTCTTCCGATCT GTCACTGTCACCATCCTCGTAGAGGA
HEK site4_off6_Adaptor_F	ACACTCTTTCCCTACACGACGCTCTTCCGATCT AGAGGCTCTAATCAAAGAGCAAGAATTTG
HEK site4_off6_Adaptor_R	GTGACTGGAGTTCAGACGTGTGCTCTTCCGATCT GGCTCTCCAAAGAAACTTGATGTTG
RNF2_on_Adaptor_F	ACACTCTTTCCCTACACGACGCTCTTCCGATCT CTAAAAATGTATCCCAGTTTACACGTCTCA
RNF2_on_Adaptor_R	GTGACTGGAGTTCAGACGTGTGCTCTTCCGATCT AGCACTTCCCTTCCAAATACTAAAATTGTT
RNF2_off1_Adaptor_F	ACACTCTTTCCCTACACGACGCTCTTCCGATCT AGACATCATCATGATAAATCTATTTGGTCT
RNF2_off1_Adaptor_R	GTGACTGGAGTTCAGACGTGTGCTCTTCCGATCT ATATTTTAAGCTAGAATGTGTTTGTTGACA
RNF2_off2_Adaptor_F	ACACTCTTTCCCTACACGACGCTCTTCCGATCT GGTTTTTGCATTACTTGGGAAGCTAGTG
RNF2_off2_Adaptor_R	GTGACTGGAGTTCAGACGTGTGCTCTTCCGATCT AGGGAAAGTTATATGCAGCCATTGTG
RNF2_off3_Adaptor_F	ACACTCTTTCCCTACACGACGCTCTTCCGATCT AGTGTGGGATAATGCTGGGGTG
RNF2_off3_Adaptor_R	GTGACTGGAGTTCAGACGTGTGCTCTTCCGATCT CAGCGTGTTCTTATGACTATTAGCAC
ZNF609 (Spacer 8A)_Adaptor_F	ACACTCTTTCCCTACACGACGCTCTTCCGATCT TGAAGCCCGCAAGGACCGAACA
ZNF609 (Spacer 8A)_Adaptor_R	GTGACTGGAGTTCAGACGTGTGCTCTTCCGATCT GCATCGTGAACATTTTTGTGTCAATTAGC
RP11-77I22.4 (Spacer 15A)_Adaptor_F	ACACTCTTTCCCTACACGACGCTCTTCCGATCT CAATATTCATGCCTTCTTTCACCTTGCC
RP11-77I22.4 (Spacer 15A)_Adaptor_R	GTGACTGGAGTTCAGACGTGTGCTCTTCCGATCT ACCTTTGGTAGTTTCCATCTATGTCGAG

Supplementary Table 4. Comparative analysis of the CRISPR amplification method and a conventional method (GUIDE-seq) for the detection of intracellular off-target mutations induced by CRISPR-Cas12a (Cpf1). PAM sequences(TTTN) of the AsCas12a effector are shown in blue and mismatched sequence to each on-target sites are shown in red respectively. (O) indicates detection, (X) indicates no detection and a question mark (?) indicates an ambiguous detection (indel frequency (%) below or near the detection limit =0.5%) with applied methods. NGS: finally confirmed by next generation sequencing. Yellow mark indicates the mutant DNA

detection with CRISPR amplification methods not by conventional NGS methods.

Target sequence (*RPL32P3*)	GUIDE-seq[1] (Reported data)	Targeted amplicon sequencing (NGS, HEK293FT)	CRISPR enrichment (NGS, HEK293FT)	CRISPR enrichment (NGS, U2OS)
***RPL32P3*-on target** (TTTGGGGTGATCAGACCCAACAGCAGG)	O	O	O	O
***RPL32P3*-off target1** (TTTGGGGTGATCAGACCCAACAcCAGG)	O	O	O	O
***RPL32P3*-off target2** (TTTGGGGTGATCAGACCCAACAcCAGG)	O	O	O	O
***RPL32P3*-off target3** (TTTGGGGTGATCAGACCCAACAcCAGG)	O	O	O	O
***RPL32P3*-off target4** (TTTGGGGTGATCAGgCCCAACAcCAGG)	O	O	O	O
***RPL32P3*-off target5** (TTTGGGGTGATCAGACCCAACccCAGG)	O	?	O	O
***RPL32P3*-off target6** (TTTGGGGTGATCAGACCtAACActAGG)	X	X	X	X

***RPL32P3*-off target7** (**TTTG**GGGTGATCcaACCCAA CAcCAGG)	**X**	**X**	**X**	**X**
***RPL32P3*-off target8** (**TTTG**GGGTGgcCAGACCCAA CAcCAGG)	**X**	**X**	**X**	**X**
***RPL32P3*-off target9** (**TTTG**GGGTGgaCAGACCCAA CAcCAGG)	**X**	**X**	**X**	**X**
***RPL32P3*-off target10** (**TTTT**GGGTGtTCAGgaCCAAC AaCAGG)	**X**	**X**	**X**	**X**

Supplementary Table 5. Comparative analysis of the CRISPR amplification method and a conventional method (targeted amplicon sequencing) for the detection of intracellular off-target mutations induced by CRISPR-Cas12a. PAM sequences (TTTN) of the AsCas12a effector are shown in blue and mismatched sequence to each on-target sites are shown in red respectively. Guide-seq numbers were presented according to the reference paper. (O) indicates detection and (X) indicates no detection with applied methods. NGS: finally confirmed by next generation sequencing.

**Target sequence (*DNMT1*-site3)**	**GUIDE-seq read counts[1] (Reported data)**	**CRISPR enrichment (NGS, HEK293FT)**
***DNMT1*-site3-on target** (**TTTC** CTGATGGTCCATGTCTGTTACTC)	**783**	**O**

***DNMT1*-site3-off target1** (**TTTC** CTGATGGTCCAcGcCTGTTAaca)	**0**	**X**
***DNMT1*-site3-off target2** (**TTTC** CTGATGGTCCATacCTGTTAaca)	**2**	**O**
***DNMT1*-site3-off target3** (**TTTC** CTGATGGTCCATGTCTGaattag)	**1174**	**O**

Supplementary Table 6. Comparative analysis of the CRISPR amplification method and a conventional method (targeted amplicon sequencing) for the detection of intracellular off-target mutations induced by CRISPR-Cas9. PAM sequences (NGG) of the SpCas9 effector are shown in blue and mismatched sequence to each on-target sites are shown in red respectively. (O) indicates detection and (X) indicates no detection with applied methods. NGS: finally confirmed by next generation sequencing. Yellow mark indicates the mutant DNA detection with CRISPR amplification methods not by conventional NGS methods.

**Target sequence** (***FAT3***)	**Targeted amplicon sequencing** (**NGS, HEK293FT**)	**CRISPR enrichment** (**NGS, HEK293FT**)
***FAT3*-on target** (GAGCTGCTTAAGCATTTCAA **GGG**)	**O**	**O**
***FAT3*-off target2** (GcAAACAAAaCAgAGACTGA **AGG**)	**X**	**X**
***FAT3*-off target3** (GTAAACAAcaCAgAGACTGA **AGG**)	**X**	**O**

***FAT3*-off target4** (GgAAACAgAGCATAGAaTGA **TGG)**	**X**	**X**

Supplementary Table 7. Comparative analysis of the CRISPR amplification method and a conventional method (targeted amplicon sequencing, NGS) for the detection of intracellular off-target mutations induced by CRISPR-Cas9. PAM sequences (NGG) of the SpCas9 effector are shown in blue and mismatched sequence to each on-target sites are shown in red respectively. Guide-seq numbers were presented according to the reference paper. (O) indicates detection and (X) indicates no detection with applied methods. Question mark (?) indicates an ambiguous detection (indel frequency (%) below or near the detection limit =0.5%). NGS: finally confirmed by next generation sequencing. Yellow mark indicates the mutant DNA detection with CRISPR amplification methods not by conventional NGS methods.

**Target sequence (HEK site4)**	**GUIDE-seq read counts[2] (Reported data)**	**Targeted amplicon sequencing (NGS, HEK293FT)**	**CRISPR enrichment (NGS, HEK293FT)**
**HEK site4 on-target** (GGCACTGCGGCTGGAGGTGG **GGG**)	**1,054**	**O**	**O**
**HEK site4 off1-target** (tGCACTGCGGCcGGAGGaGG **TGG**)	**2,475**	**O**	**O**
**HEK site4 off2-target** (GGCAtcaCGGCTGGAGGTGG **AGG**)	**1,097**	**?**	**O**
**HEK site4 off3-target** (aGCAgTGCGGCTaGAGGTGG **TGG**)	**981**	**O**	**O**

**HEK site4 off4-target** (GGCACTGaGaaaGGAGGTGG **AGG**)	**13**	**X**	**X**
**HEK site4 off5-target** (GGgcaTGCGGCTGGAaGTGG **TGG**)	**3**	**?**	**O**
**HEK site4 off6-target** (GCACaGgGGCTGGAGGTGG **GGC**)	**3**	**X**	**X**

Supplementary Table 8. Comparative analysis of the CRISPR amplification method and a conventional method (targeted amplicon sequencing, NGS) for the detection of intracellular off-target mutations induced by CRISPR-Cas9. PAM sequences (NGG) of the SpCas9 effector are shown in blue and mismatched sequence to each on-target sites are shown in red respectively. Guide-seq numbers were presented according to the reference paper. (O) indicates detection and (X) indicates no detection with applied methods. NGS: finally confirmed by next generation sequencing.

**Target sequence (*RNF2*)**	**GUIDE-seq read counts[2] (Reported data)**	**Targeted amplicon sequencing (NGS, HEK293FT)**	**CRISPR enrichment (NGS, HEK293FT)**
***RNF2* on-target** (GTCATCTTAGTCATTACCTG **AGG**)	**6,643**	**O**	**O**
***RNF2* off1-target** (GgtATCTaAGTCATTACCTG **TGG**)	**0**	**X**	**X**
***RNF2* off2target** (GTaATCTgAGTCATTtCCTG **GGG**)	**0**	**X**	**X**

***RNF2* off3target**<br>(GTCATCcTAGTgcTTACCTG **AGG**)	**0**	**X**	**X**

Supplementary Table 9. Comparative analysis of the CRISPR amplification method and a conventional method (targeted amplicon sequencing) for the detection of intracellular off-target mutations induced by ABE. PAM sequences (NGG) of the adenine base editor are shown in blue and mismatched sequence to each on-target sites are shown in red respectively. (O) indicates detection and (X) indicates no detection with applied methods. NGS: finally confirmed by next generation sequencing. Yellow mark indicates the mutant DNA detection with CRISPR amplification methods not by conventional NGS methods.

**Target sequence**<br>**(*PSMB2*)**	**Targeted amplicon sequencing**<br>**(NGS, HEK293FT)**	**CRISPR enrichment**<br>**(NGS, HEK293FT)**
***PSMB2*-on target**<br>(GTAAACAAAGCATAGACTGA **GGG**)	**O**	**O**
***PSMB2*-off target2**<br>(GcAAACAAAaCAgAGACTGA **AGG**)	**X**	**O**
***PSMB2*-off target3**<br>(GTAAACAAcaCAgAGACTGA **AGG**)	**X**	**O**
***PSMB2*-off target4**<br>(GgAAACAgAGCATAGAaTGA **TGG**)	**X**	**O**

Supplementary Figures

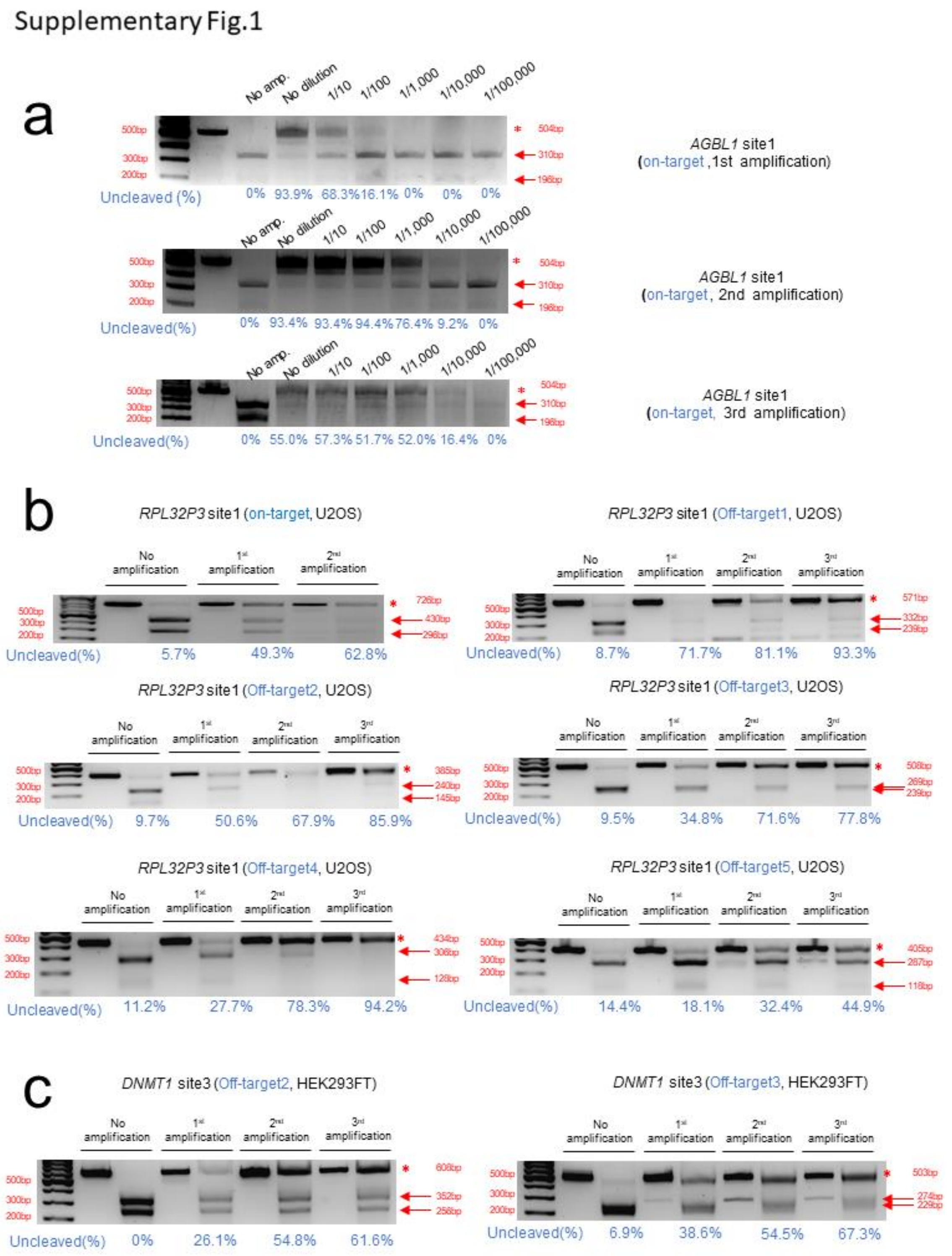

Supplementary Figure 1. Genotyping of the Cas12a induced mutant DNA enrichment with CRISPR amplification. (a) Relatively compare the results of serially diluted (from 1X to 1/ 100000X) each genomic DNA sample with mutations induced by Cas12a. Relative mutant DNA frequency (%) at *AGBL1* locus was calculated by cleaving DNA amplicons with optimally designed crRNA for Cas12a. The cleaved amplicons were separated by 2% agarose gel. **(b)** Relative mutant DNA frequency (%) for predicted off-target sites of *RPL32P3* site in genomic DNA from U2OS cells. **(c)** Relative mutant DNA frequency (%) for predicted off-target sites of *DNMT1* in genomic DNA from U2OS cells. Uncleaved DNA fractions are indicated by asterisk and cleaved DNA fractions are indicated by red arrows, respectively. Uncleaved mutant DNA frequency (%) was calculated by intensity ratio of band patterns (uncleaved fraction (%) / cleaved fraction (%) + uncleaved fraction (%)). Representative gel image was shown from two (N=2) independent experiments. Source data are provided as a Source Data file.

Supplementary Fig.2

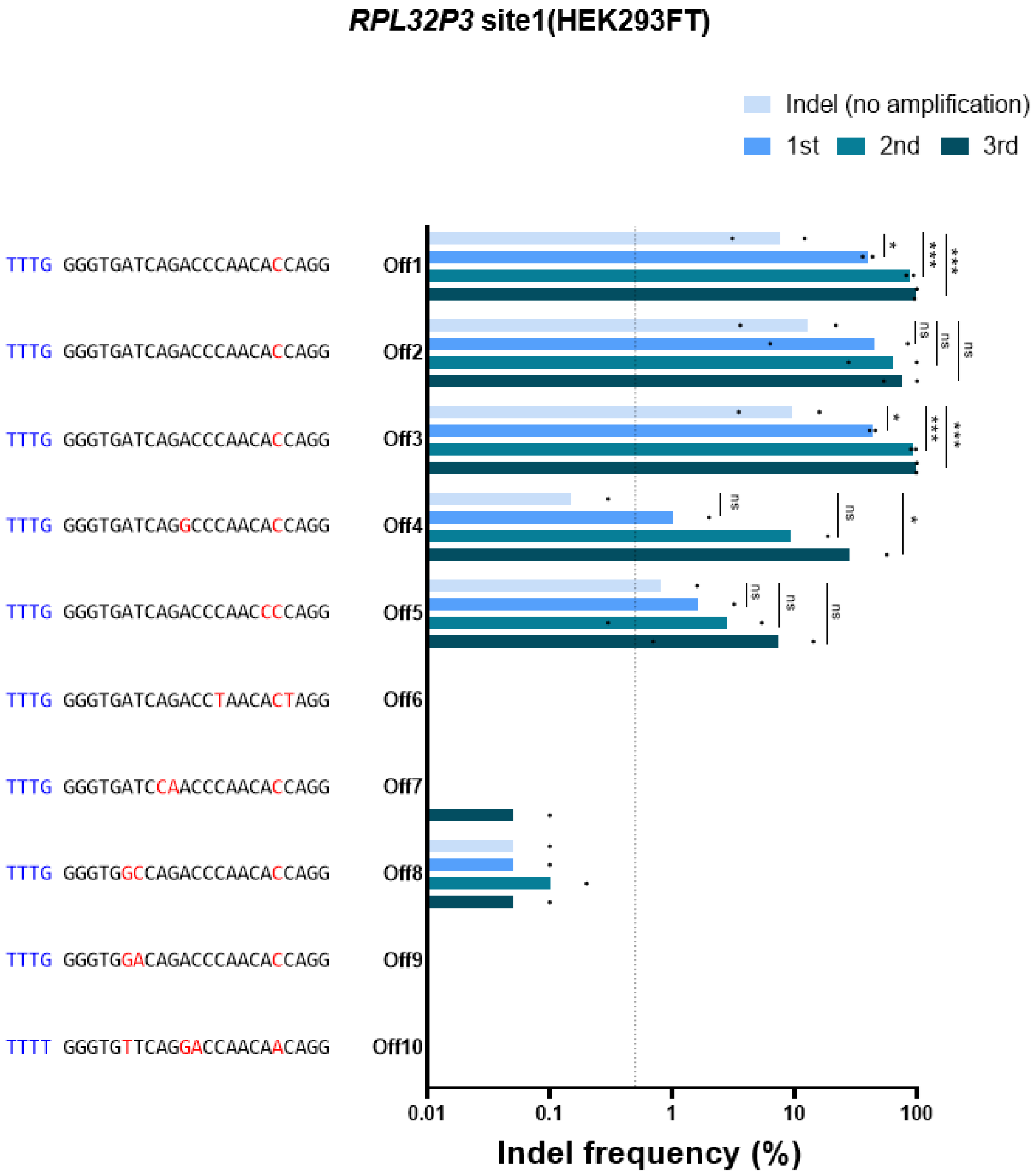

Supplementary Figure 2. Detection of the intracellular off-target mutation induced by CRISPR-Cas12a (Cpf1) by using CRISPR amplification. Detection of off-target mutations for the target sequence (*RPL32P3* locus) generated by CRISPR-Cas12a effector in HEK293FT cells. PCR amplicons were generated for 10 off-target sequences same with **(Fig. 2)** and indel frequency (%) was analyzed by next-generation sequencing after multiple CRISPR amplification. The Y axis represents the amplified target and off-target sequences (The PAM sequence and mismatch to wild-

type reference in protospacer is shown in blue and red color, respectively), and the X axis represents the frequency (%) of indels on a log scale. The dashed line indicates the NGS detection limit (=0.5%). Each amplification stage for mutant DNA enrichment is shown in light blue (no amplification), blue (1st CRISPR amplification), green (2nd CRISPR amplification) and dark green (3rd CRISPR amplification). Data are shown as mean from two (N=2) independent experiments. *P*-values are calculated using a one-way ANOVA, Tukey's test (ns: not significant, *P**=0.0332, *P***=0.0021, *P****=0.0002, *P*****=0.0001).

Supplementary Fig.3

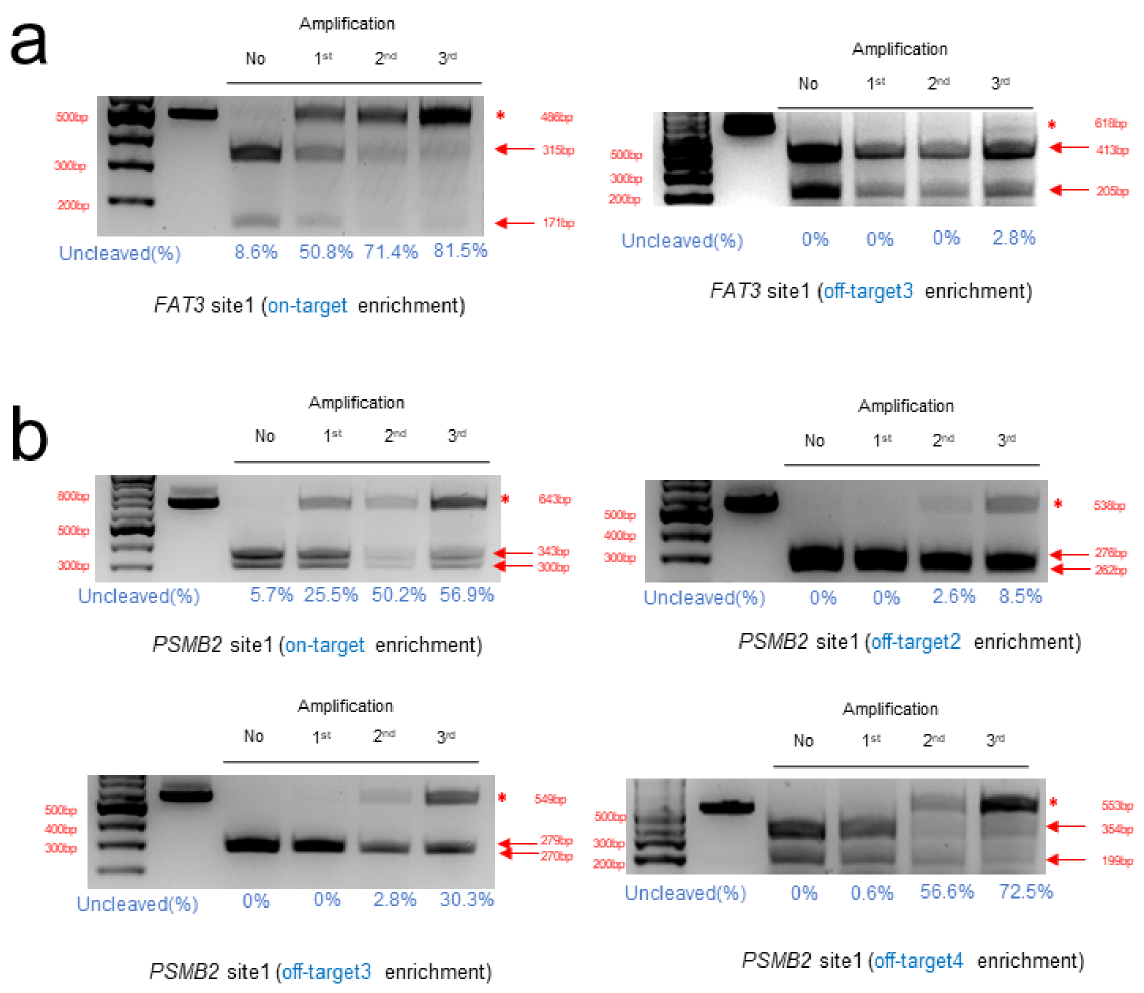

Supplementary Figure 3. Genotyping of the CRISPR amplification of intracellular off-target mutations induced by CRISPR-Cas9 and adenine base editor (ABE). (a) The relative mutant DNA frequency (%) for target and predicted Cas9 off-target sites of *FAT3* sequence in genomic DNA from HEK293FT cell is calculated by cleavage

and separation of PCR amplicons on 2% agarose gel. **(b)** The relative single-base substituted DNA frequency (%) for target and predicted base editor off-target sites of *PSMB2* sequence in genomic DNA from HEK293FT cell is calculated by cleavage and separation of PCR amplicons on 2% agarose gel. Uncleaved DNA fractions are indicated by asterisk and cleaved DNA fractions are indicated by red arrows. Uncleaved mutant DNA frequency (%) was calculated by intensity ratio of band patterns (uncleaved fraction (%) / cleaved fraction (%) + uncleaved fraction (%)). Representative gel image was shown from two (N=2) independent experiments. Source data are provided as a Source Data file.

Supplementary Fig.4

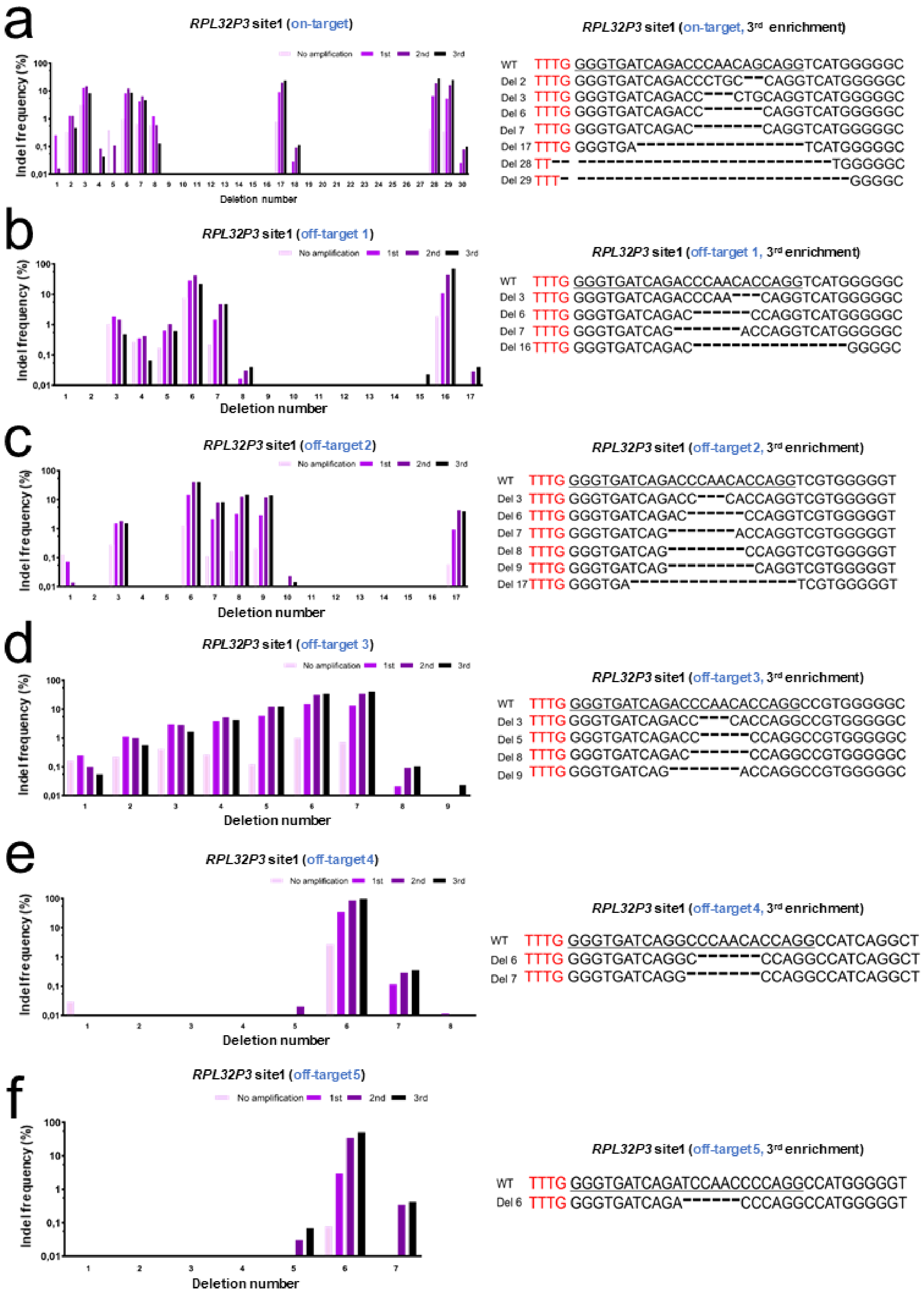

Supplementary Figure 4. Enriched mutant DNA pattern induced by Cas12a at predicted off-target sites for *RPL32P3* target sequence. NGS data analysis of AsCas12a induced indel patterns enriched by multiple round CRISPR amplification for **(a)** *RPL32P3* on-target site**, (b)** off-target site1, **(c)** off-target site2, **(d)** off-target site3, **(e)** off-target site4, and **(f)** off-target site5, respectively. Each amplification stage for mutant DNA enrichment is shown in pink (no amplification), light purple (1st CRISPR amplification), purple (2nd CRISPR amplification) and black (3rd CRISPR amplification).

Supplementary Fig.5

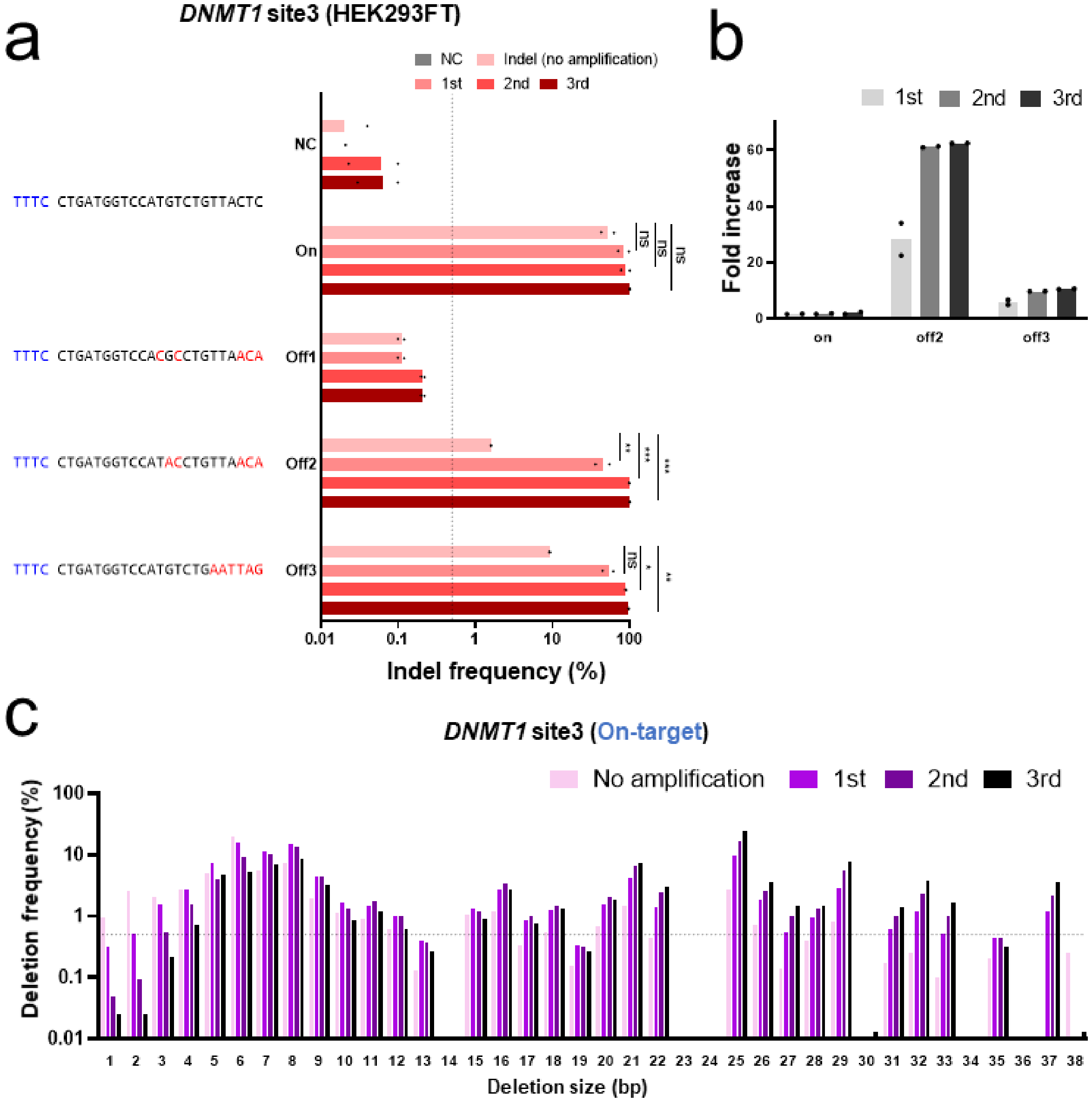

***DNMT1* site3 (On-target, 3rd enrichment)**

```
WT      ATGTTTC CTGATGGTCCATGTCTGTTACTCGCCTGTCAAGTGGCGT
Del 5   ATGTTTC CTGATGGTCCATG-----TACTCGCCTGTCAAGTGGCGT
Del 6   ATGTTTC CTGATGGTCCATG------ACTCGCCTGTCAAGTGGCGT
Del 7   ATGTTTC CTGATGGTCCA-------TACTCGCCTGTCAAGTGGCGT
Del 8   ATGTTTC CTGATGGTCCAT--------CTCGCCTGTCAAGTGGCGT
Del 9   ATGTTTC CTGATGGTCCATG---------CGCCTGTCAAGTGGCGT
Del 16  ATGTTTC CTGATGGT----------------CCTGTCAAGTGGCGT
Del 21  ATGTTTC CTGATG---------------------GTCAAGTGGCGT
Del 22  ATGTTTC CTGA----------------------TGTCAAGTGGCGT
Del 25  ATGTTT- ------------------------CCTGTCAAGTGGCGT
Del 26  ATGTT-- ------------------------CCTGTCAAGTGGCGT
Del 27  ATGTTTC CTGAT---------------------------GTGGCGT
Del 28  ATGTTTC CTGATGGT----------------------------CGT
Del 29  ATGTTTC CTGA-----------------------------TGGCGT
Del 31  ATGTTTC CTGATG-------------------------------GT
Del 32  A------ --------------------------TGTCAAGTGGCGT
Del 33  ATGT--- ------------------------------AAGTGGCGT
Del 37  ATGTTTC C-------------------------------------T
```

Supplementary Fig.5

d

***DNMT1* site3 (Off-target 2)**

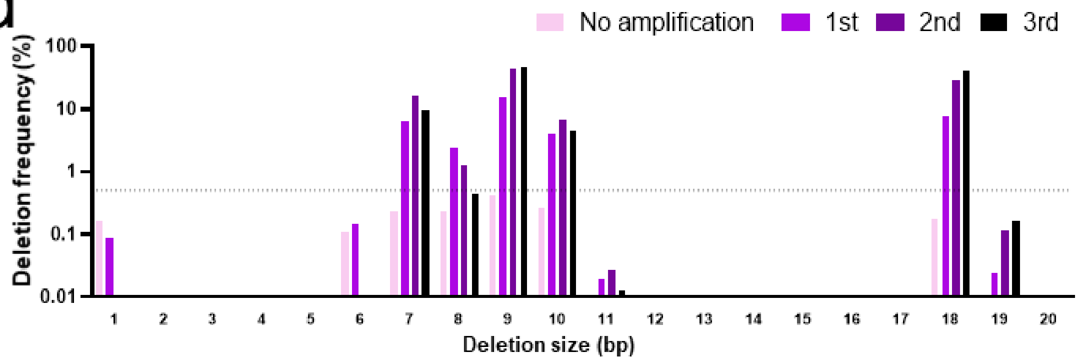

***DNMT1* site3 (Off-target 2, 3rd enrichment)**

```
WT      TTTC CTGATGGTCCATACCTGTTAACATTAAAATGTC
Del 7   TTTC CTGATGGTCCA-------TAACATTAAAATGTC
Del 9   TTTC CTGATGGTCCAT---------CATTAAAATGTC
Del 10  TTTC CTGATGGTCCAT----------ATTAAAATGTC
Del 18  TTTC CTGAT------------------TTAAAATGTC
```

e

***DNMT1* site3 (Off-target 3)**

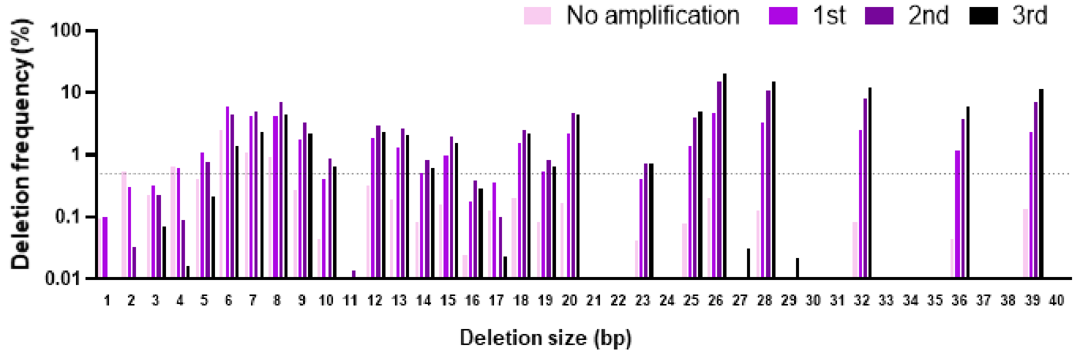

***DNMT1* site3 (Off-target 3, 3rd enrichment)**

```
WT      TTTC CTGATGGTCCATGTCTGAATTAGACACCCCTCTTCT
Del 6   TTTC CTGATGGTCCATG------TTAGACACCCCTCTTCT
Del 7   TTTC CTGATGGTCCAT-------TTAGACACCCCTCTTCT
Del 8   TTTC CTGATGGTCC--------ATTAGACACCCCTCTTCT
Del 9   TTTC CTGATGGTCCA---------TAGACACCCCTCTTCT
Del 12  TTTC CTGATGGTCCAT------------CACCCCTCTTCT
Del 13  TTTC CTGATG-------------TTAGACACCCCTCTTCT
Del 18  TTTC CTGATGGT------------------CCCCTCTTCT
Del 20  TTTC CT--------------------GACACCCCTCTTCT
Del 25  TTTC C-------------------------CCCCTCTTCT
Del 26  TTTC --------------------------CCCCTCTTCT
Del 28  TT-- ------------------------------TCTTCT
Del 32  TTT- ---------------------------CCCTCTTCT
Del 36  ---- --------------------------------TTCT
Del 39  ---- -----------------------------------T
```

Supplementary Figure 5. Enriched mutant DNA pattern induced by Cas12a at

predicted off-target sites for *DNMT1* target sequence. (a) Detection of off-target mutations for the target sequence (*DNMT1*) generated by the CRISPR-Cas12a effector in HEK293FT cells. PCR amplicons were generated for on-target and three off-target sequences (The PAM sequence and mismatch to wild-type reference in protospacer is shown in blue and red color, respectively) predicted *in silico* and the indel frequency (%) was analyzed by NGS after sequential CRISPR amplifications. NC indicates a negative control for no Cas12a delivery into the cells. Each amplification stage for mutant DNA enrichment is shown in gray (NC), light pink (no amplification), pink (1st CRISPR amplification), red (2nd CRISPR amplification) and dark red (3rd CRISPR amplification). The dashed line indicates the NGS detection limit (=0.5%). Data are shown as mean from two (N=2) independent experiments. *P*-values are calculated using a one-way ANOVA, Tukey's test (ns: not significant, *P**=0.0332, *P***=0.0021, *P****=0.0002, *P*****=0.0001). **(b)** Fold increases in *DNMT1* target and off-target mutant DNA after CRISPR amplification (N=2). **(c-e)** NGS analysis of AsCas12a induced indel patterns on *DNMT1*-site3 locus enriched by third round CRISPR amplification for **(c)** on-target**, (d)** off-target site2**, (e)** off-target site3, respectively. Each amplification stage for mutant DNA enrichment is shown in pink (no amplification), light purple (1st CRISPR amplification), purple (2nd CRISPR amplification) and black (3rd CRISPR amplification).

Supplementary Fig.6

a

***FAT3* site1 (On-target)**

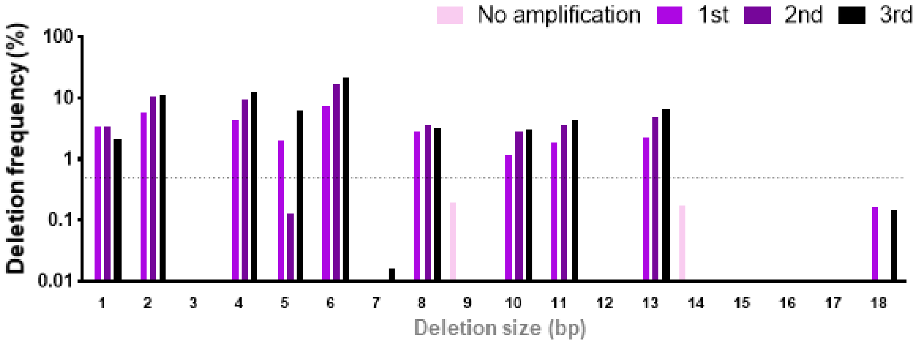

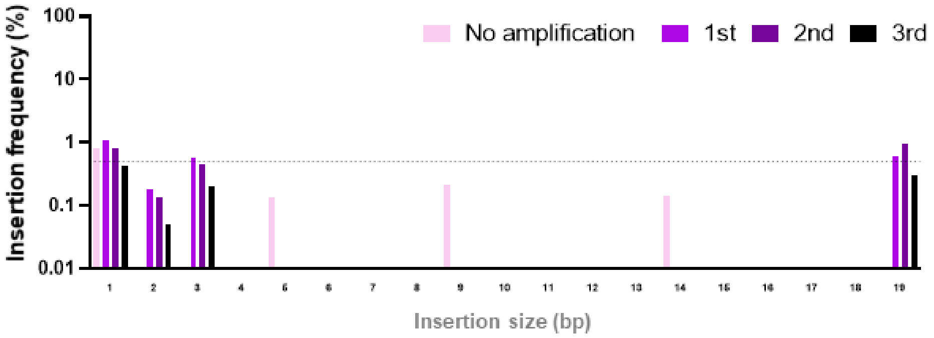

***FAT3* site1**
(On-target, 3rd enrichment, deletion pattern)

```
WT      TTTGGAGCTGCTTAAGCATTTCAAGGGAAGAAACCCT
Del 1   TTTGGAGCTGCTTAAGCATT-CAAGGGAAGAAACCCT
Del 2   TTTGGAGCTGCTTAAGCAT--CAAGGGAAGAAACCCT
Del 4   TTTGGAGCTGCTTAAGC----CAAGGGAAGAAACCCT
Del 5   TTTGGAGCTGCTTAAG-----CAAGGGAAGAAACCCT
Del 6   TTTGGAGCTGCTTAAGC------AGGGAAGAAACCCT
Del 8   TTTGGAGCTGCTT--------CAAGGGAAGAAACCCT
Del 10  TTTGGAGCTGCTTAA----------GGAAGAAACCCT
Del 11  TTTGGAGCTGCTTAAGCATTTC-----------CCCT
Del 13  TTTGGAGC-------------CAAGGGAAGAAACCCT
```

***FAT3* site1**
(On-target, 3rd enrichment, insertion pattern)

```
WT      TTTGGAGCTGCTTAAGCATTT---CAA  GGGAAGA
Ins 1   TTTGGAGCTGCTTAAGCATTTA--CAA  GGGAAGA
Ins 2   TTTGGAGCTGCTTAAGCATTTTT-CAA  GGGAAGA
Ins 3   TTTGGAGCTGCTTAAGCATTTTTTCAA  GGGAAGA
```

b

***FAT3* site1 (Off-target 3)**

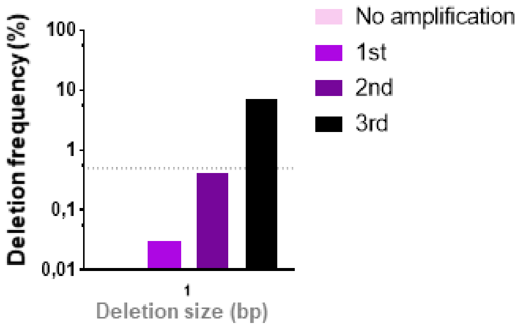

***FAT3* site1**
(Off-target 3, 3rd enrichment, deletion pattern)

```
WT      CCCTGGAGATGCAGAAGCATTTCAAGGGGACAGCCAC
Del 1   CCCTGGAGATGCAGAAGCATT-CAAGGGGACAGCCAC
```

Supplementary Figure 6. Enriched mutant DNA pattern induced by Cas9 effector

at predicted off-target sites for *FAT3* target sequence. NGS analysis of SpCas9 induced indel patterns on *FAT3* locus enriched by third round CRISPR amplification for **(a)** on-target and **(b)** off-target site3. PAM sequence (NGG) for SpCas9 is shown in red color and inserted DNA bases are shown in orange color respectively. Each amplification stage for mutant DNA enrichment is shown in pink (no amplification, indel only), light purple (1st CRISPR amplification), purple (2nd CRISPR amplification) and black (3rd CRISPR amplification).

Supplementary Fig.7

a

HEK293 site4 (On-target)

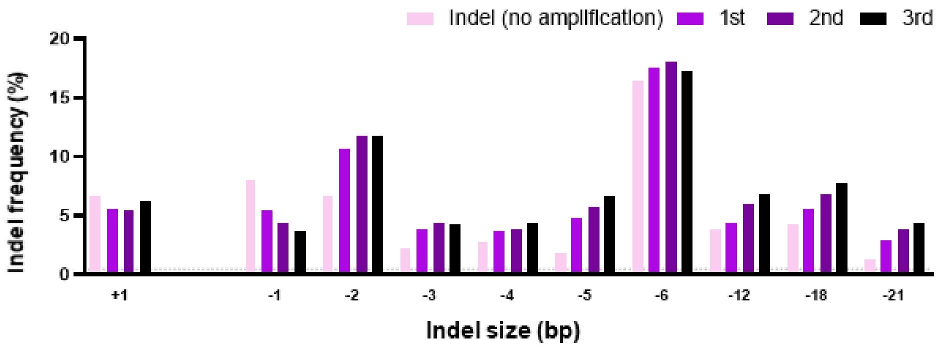

HEK293 site4 (On-target, 3rd enrichment, deletion pattern)

```
WT      GGTGGCACTGCGGCTGGAGGTGG GGGTTAAAG
Del 1   GGTGGCACTGCGGCTGGAG-TGG GGGTTAAAG
Del 2   GGTGGCACTGCGGCTGGA--TGG GGGTTAAAG
Del 3   GGTGGCACTGCGGCTGG---TGG GGGTTAAAG
Del 4   GGTGGCACTGCGGCTG----TGG GGGTTAAAG
Del 5   GGTGGCACTGCGGCTGGA----- GGGTTAAAG
Del 6   GGTGGCACTGCGGC------TGG GGGTTAAAG
Del 6   GGTGGCACTGCGGCTGGA----- -GGTTAAAG
Del 12  GGTGGCACTG------------G GGGTTAAAG
Del 13  GGTGGCA-------------TGG GGGTTAAAG
Del 18  GGTGGCAC--------------- ---TTAAAG
Del 21  ---------------------GG GGGTTAAAG
```

b

HEK293 site4 (Off-target 1)

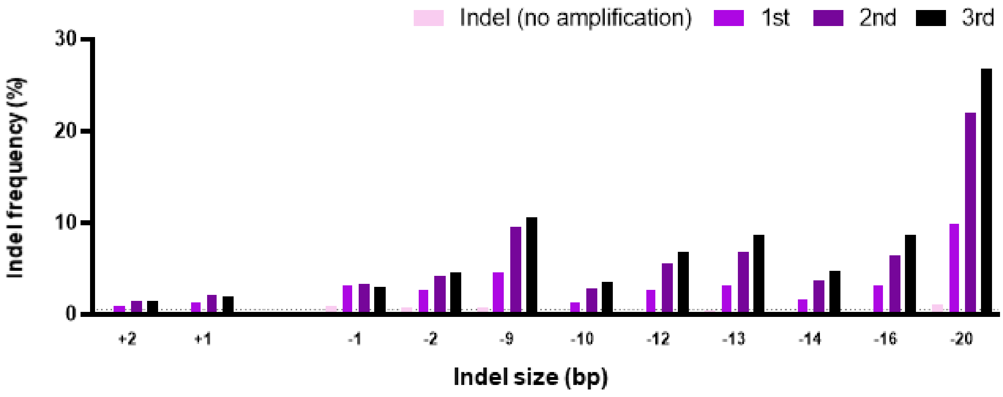

HEK293 site4 (Off-target 1, 3rd enrichment, deletion pattern)

```
WT      GGTGCACTGCGGCCGGAGGAGG TGGAGGATGGA
Del 1   GGTGCACTGCGGCCGGAGG-GG TGGAGGATGGA
Del 2   GGTGCACTGCGGCCGGA--AGG TGGAGGATGGA
Del 9   GGTGCACTGCGGCC-------- -GGAGGATGGA
Del 10  GGTGCACTGC----------GG TGGAGGATGGA
Del 12  GGTGCACT------------GG TGGAGGATGGA
Del 13  GGTGCA-------------AGG TGGAGGATGGA
Del 14  GGTGC--------------AGG TGGAGGATGGA
Del 16  GGTGCACTGCGGCC-------- --------GGA
Del 20  --------------------GG TGGAGGATGGA
```

c

HEK293 site4 (Off-target 2)

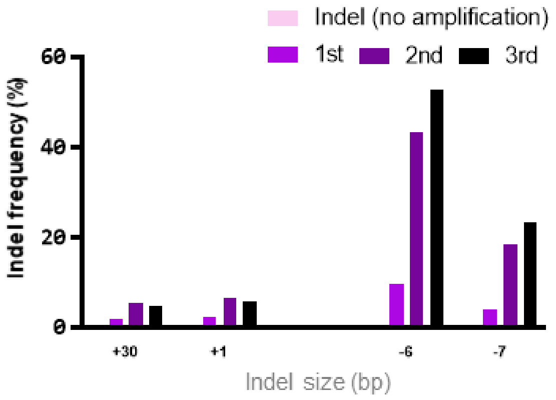

HEK293 site4
(Off-target 2, 3rd enrichment, deletion pattern)

```
WT     GGCATCACGGCTGGAGGTGG AGG
Del 6  GGCATCACGGC------TGG AGG
Del 7  GGCATCAC-------GGTGG AGG
```

HEK293 site4
(Off-target 2, 3rd enrichment, insertion pattern)

```
WT      CAAGGCATCACGGCTGGAGG------------------------------TGG AGG
Ins 1   CAAGGCATCACGGCTGGAGGA-----------------------------TGG AGG
Ins 30  CAAGGCATCACGGCTGGAGGGAACAAGTTGAAAGACCCAAGCAACCTGAATGG AGG
```

d

HEK293 site4 (Off-target 3)

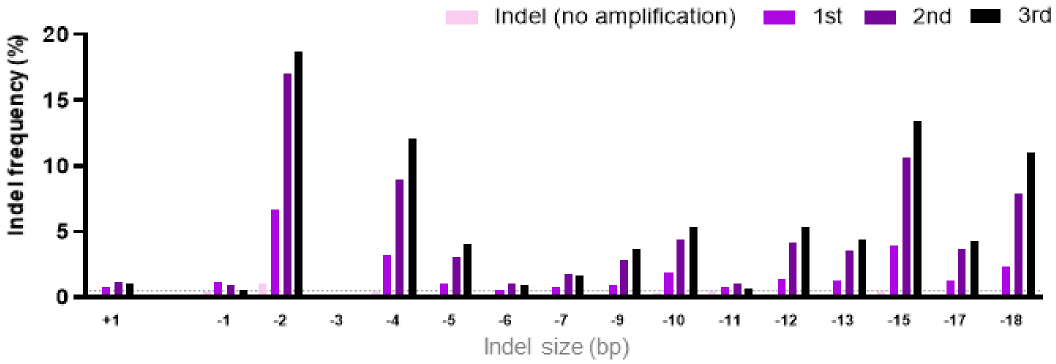

HEK293 site4 (Off-target 3, 3rd enrichment, deletion pattern)

```
WT      AGAAGCAGTGCGGCTAGAGGTGG TGGCTGGGC
Del 2   AGAAGCAGTGCGGCTAGA--TGG TGGCTGGGC
Del 4   AGAAGCAGTGCGGCTA----TGG TGGCTGGGC
Del 5   AGAAGCAGTGCGGCT-----TGG TGGCTGGGC
Del 9   AGAAGCAGTGC---------TGG TGGCTGGGC
Del 12  AGAAGCA------------GTGG TGGCTGGGC
Del 13  AGAAGCAGTGC------------ -GGCTGGGC
Del 15  AGAAGCA---------------G TGGCTGGGC
Del 17  AGAA-----------------GG TGGCTGGGC
Del 18  AGAAGCAGTGC------------ ------GGC
```

e

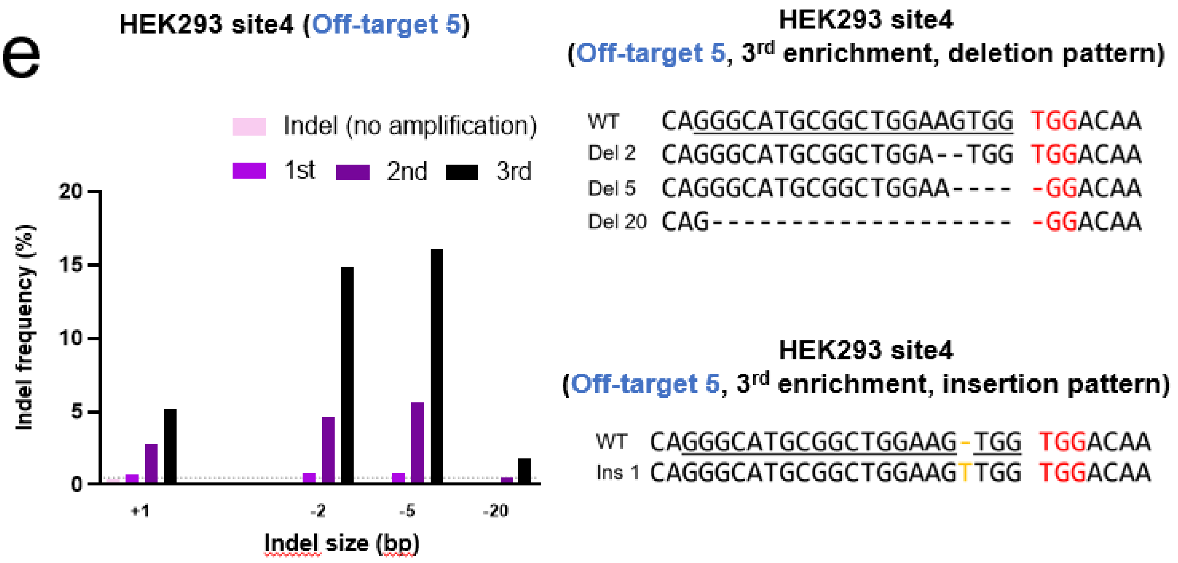

Supplementary Figure 7. Enriched mutant DNA pattern induced by CRISPR-Cas9 effector at predicted off-target sites for HEK293 site4 target sequence. NGS analysis of SpCas9 induced indel patterns on HEK293 site4 locus enriched by third round CRISPR amplification for **(a)** on-target, **(b)** off-target site1, **(c)** off-target site2, **(d)** off-target site3 and **(e)** off-target site5. PAM sequence (NGG) for SpCas9 is shown in red color. Deleted and inserted DNA bases are shown in dashed line and yellow color, respectively. Each amplification stage for mutant DNA enrichment is shown in pink (no amplification, indel only), light purple (1st CRISPR amplification), purple (2nd CRISPR amplification) and black (3rd CRISPR amplification).

Supplementary Fig.8

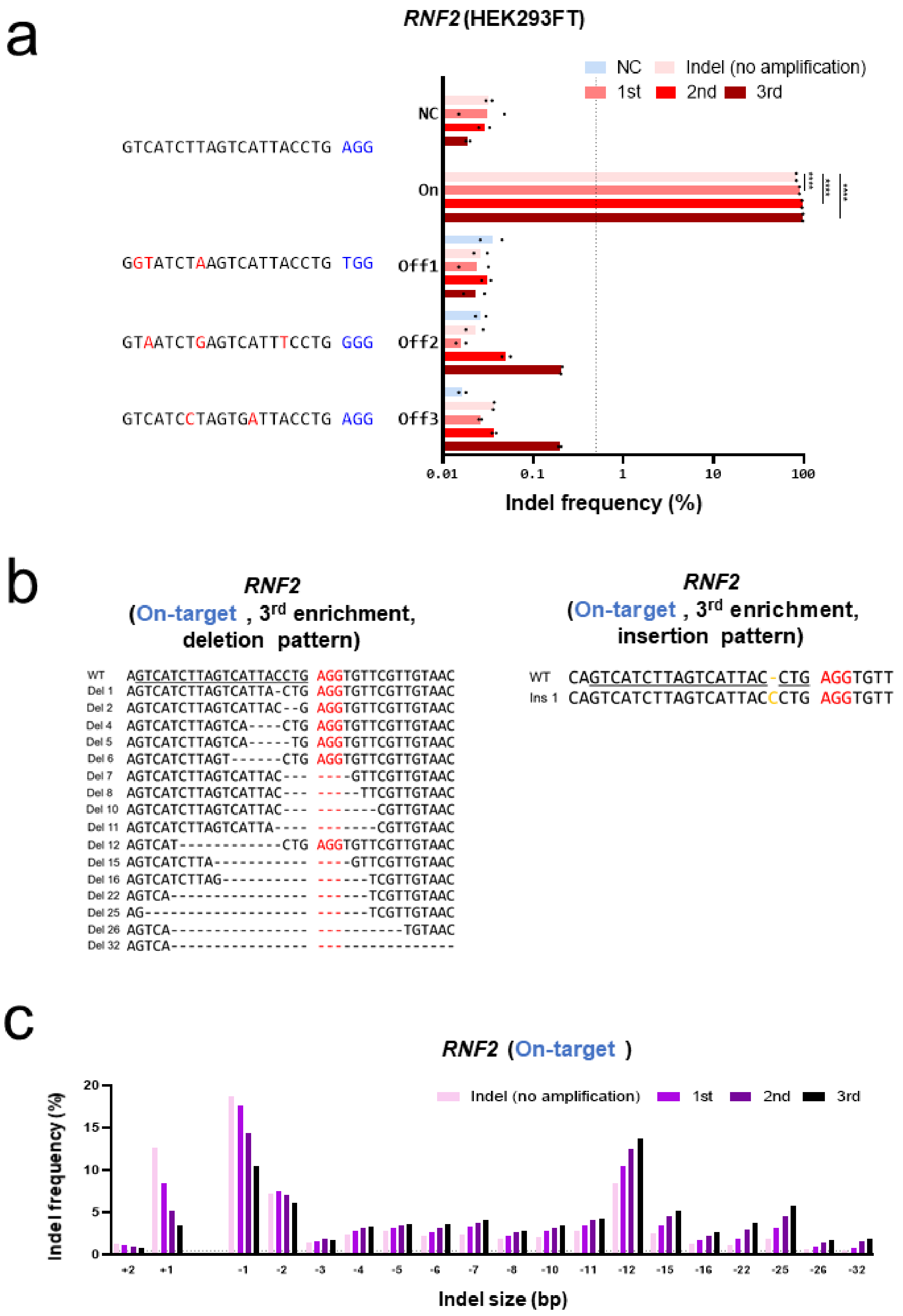

Supplementary Figure 8. Target-specific genome editing and enriched mutant DNA pattern induced by CRISPR-Cas9 effector at predicted off-target sites for

RNF2* target sequence. (a)** Detection of off-target mutations for the target sequence (*RNF2*) generated by the CRISPR-Cas9 effector in HEK293FT cells. PCR amplicons were generated for on-target and three off-target sequences (The PAM sequence and mismatch to wild-type reference in protospacer is shown in blue and red color, respectively) predicted *in silico* and the indel frequency (%) was analyzed by NGS after sequential CRISPR amplifications. NC indicates a negative control for no Cas9 delivery into the cells. Each amplification stage for mutant DNA enrichment is shown in blue (NC), light pink (no amplification, only indel), pink (1st CRISPR amplification), red (2nd CRISPR amplification) and dark red (3rd CRISPR amplification). The dashed line indicates the NGS detection limit (=0.5%). Data are shown as mean from two (N=2) independent experiments. *P*-values are calculated using a one-way ANOVA, Tukey's test (ns: not significant, *P**=0.0332, *P=0.0021, *P****=0.0002, *P*****=0.0001). **(b, c)** NGS analysis of SpCas9 induced indel patterns on *RNF2* locus enriched by third round CRISPR amplification for on-target. PAM sequence (NGG) for SpCas9 is shown in red color. Deleted and inserted DNA bases are shown in dashed line and yellow color, respectively. Each amplification stage for mutant DNA enrichment is shown in pink (no amplification, indel only), light purple (1st CRISPR amplification), purple (2nd CRISPR amplification) and black (3rd CRISPR amplification).

Supplementary Fig.9

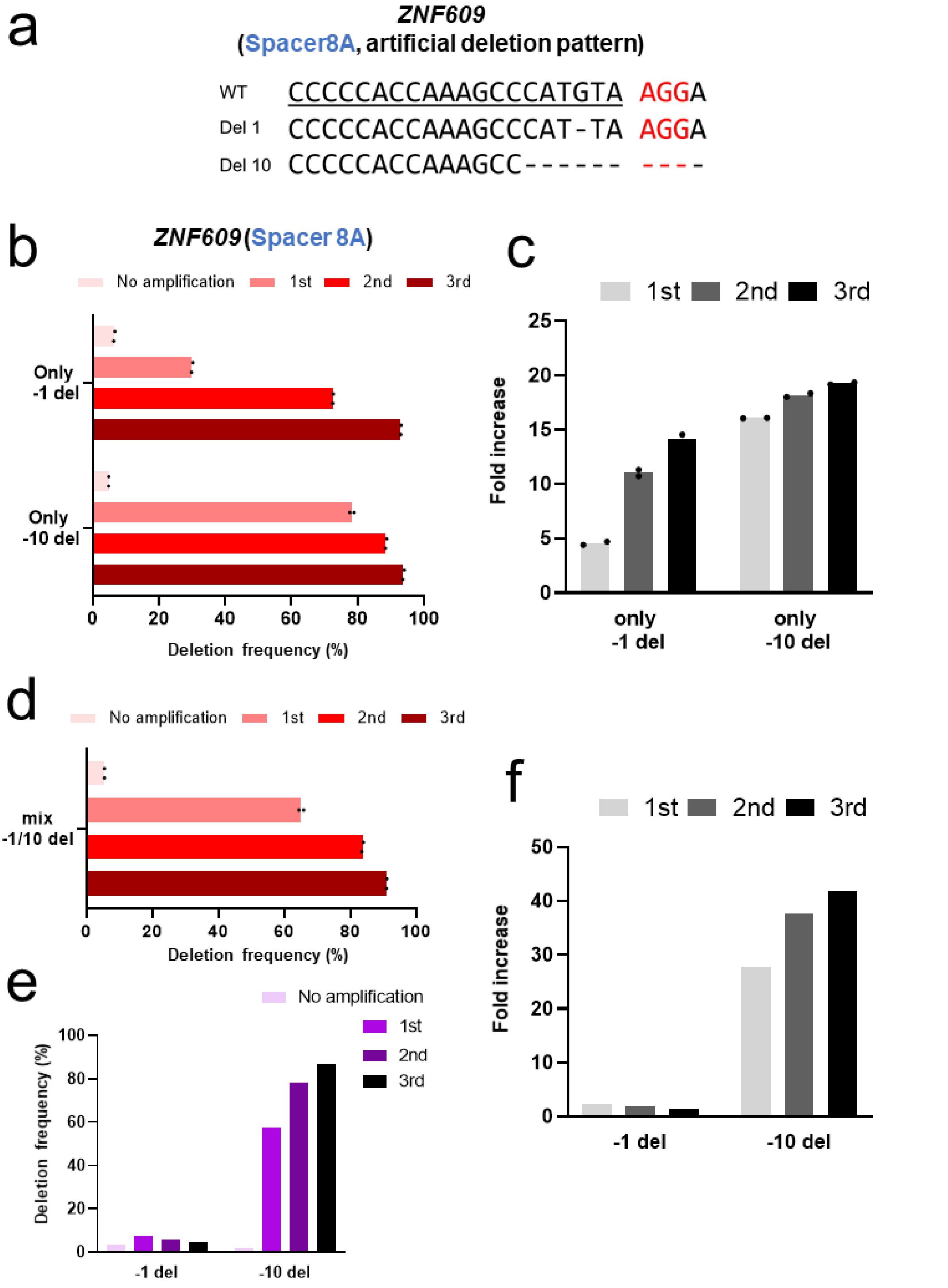

Supplementary Figure 9. Enrichment of the artificially synthesized mutant DNA amplicon with 1bp and 10bp deletions at individual or mixed condition. (a) PCR

amplicons were artificially generated for wild-type (*ZNF609*) and two deleted sequences (Del1, Del10). **(b)** Deletion frequency (%) was analyzed by NGS after sequential CRISPR amplifications with wild-type-Del 1 and wild type-Del 10 amplicon mixture, respectively. Each amplification stage for mutant DNA enrichment is shown in light pink (no amplification), pink (1st CRISPR amplification), red (2nd CRISPR amplification) and dark red (3rd CRISPR amplification). Data are shown as mean from two independent experiments (N=2). **(c)** Fold increase after CRISPR amplification (N=2) for each Del 1 and Del 10 sequence. Primary, secondary, and tertiary CRISPR amplification results are shown in gray, dark gray, and black, respectively. All experiments were conducted at least two times. **(d)** Deletion frequency (%) was analyzed by NGS after sequential CRISPR amplifications with wild-type-Del1-Del10 amplicon mixture. Each amplification stage for mutant DNA mixture enrichment is shown as (b). **(e)** Deletion frequency (%) of each Del 1 and Del 10 pattern was analyzed by NGS after sequential CRISPR amplifications. **(f)** Fold increase after CRISPR amplification (N=2) for each Del 1 and Del 10 sequence from mixed enrichment result. Data is shown as (c).

Supplementary Fig.10

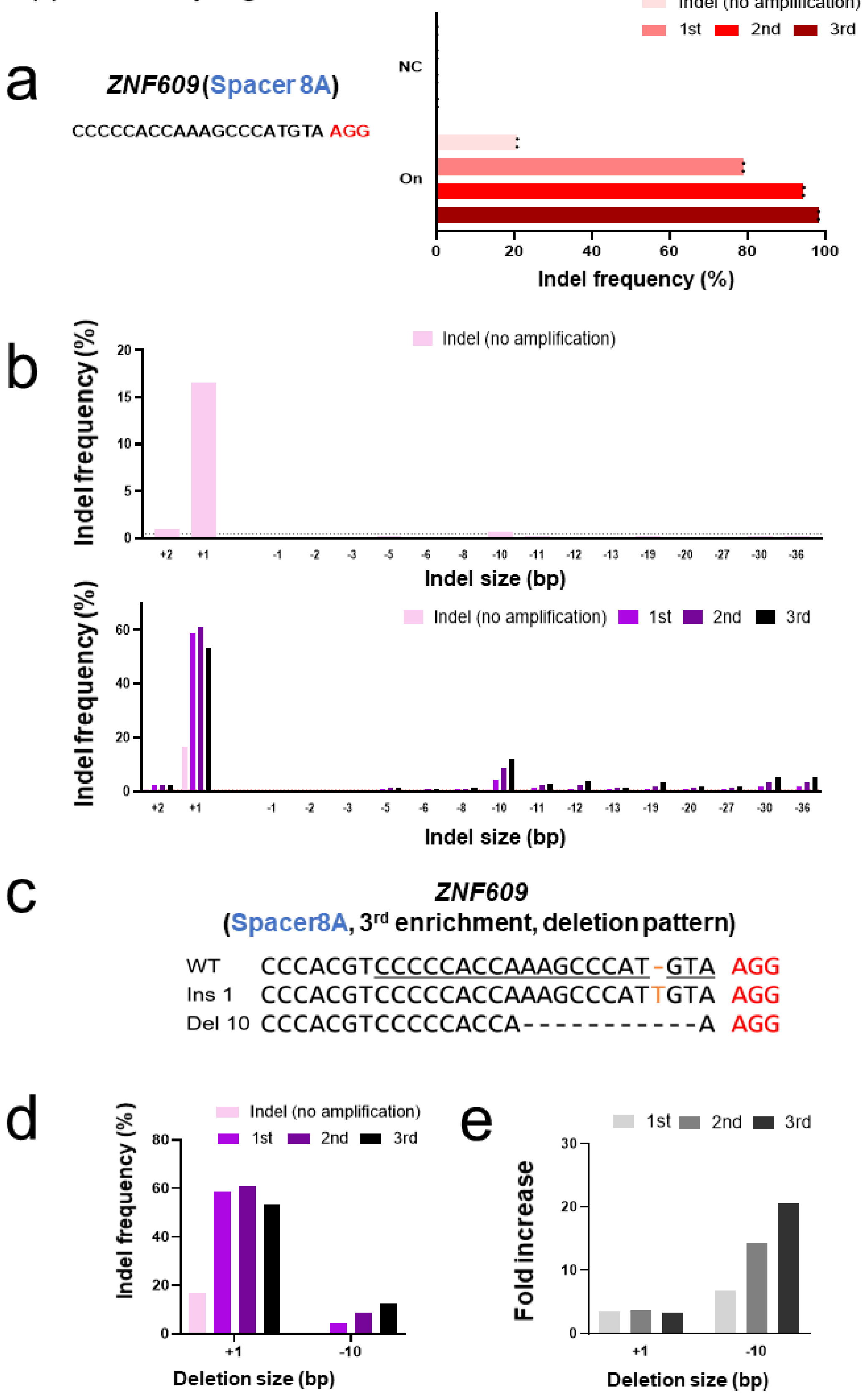

Supplementary Figure 10. Enriched mutant DNA pattern induced by Cas9 effector at *ZNF609* (Spacer8A) target sequence. (a) Detection of on-target

mutations for the target sequence (*ZNF609,* Spacer8A) generated by the CRISPR-Cas9 effector in HEK293FT cells. Indel frequency (%) was analyzed by NGS after sequential CRISPR amplifications. Each amplification stage for mutant DNA enrichment is shown in light pink (no amplification, only indel), pink (1st CRISPR amplification), red (2nd CRISPR amplification) and dark red (3rd CRISPR amplification). Data are shown as mean from two (N=2) independent experiments**.** NC indicates negative control of no Cas9 delivery into cells. **(b)** NGS analysis of CRISPR-Cas9 induced indel frequency on *ZNF609* (Spacer8A) locus. Top: Indel frequency versus various size of indel patterns from NGS sequencing data (a). Bottom: Each mutation frequency of amplification stage for various indel enrichment is shown in pink (no amplification, indel only), light purple (1st CRISPR amplification), purple (2nd CRISPR amplification) and black (3rd CRISPR amplification). **(c)** NGS analysis of CRISPR-Cas9 induced indel patterns on *ZNF609* locus enriched by third round CRISPR amplification. PAM sequence (NGG) for SpCas9 is shown in red color. Deleted and inserted DNA bases are shown in dashed line and yellow color, respectively. **(d)** Magnified view of amplified mutation frequency of 1bp insertion and 10bp deletion. **(e)** Fold increases in *ZNF609* target for 1bp insertion and 10bp deletion mutant DNA after CRISPR amplification (N=2).

Supplementary Fig.11

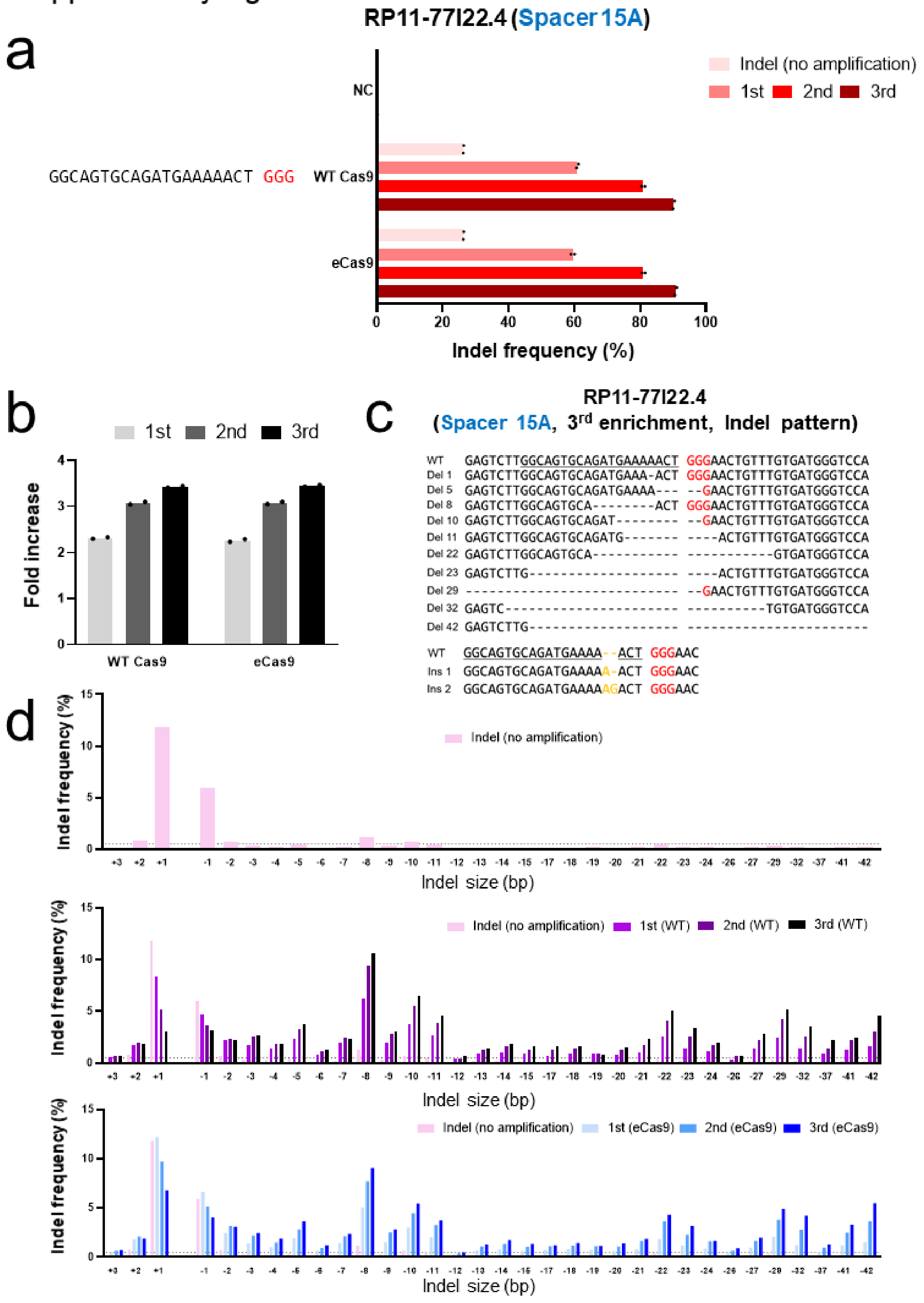

Supplementary Fig.11

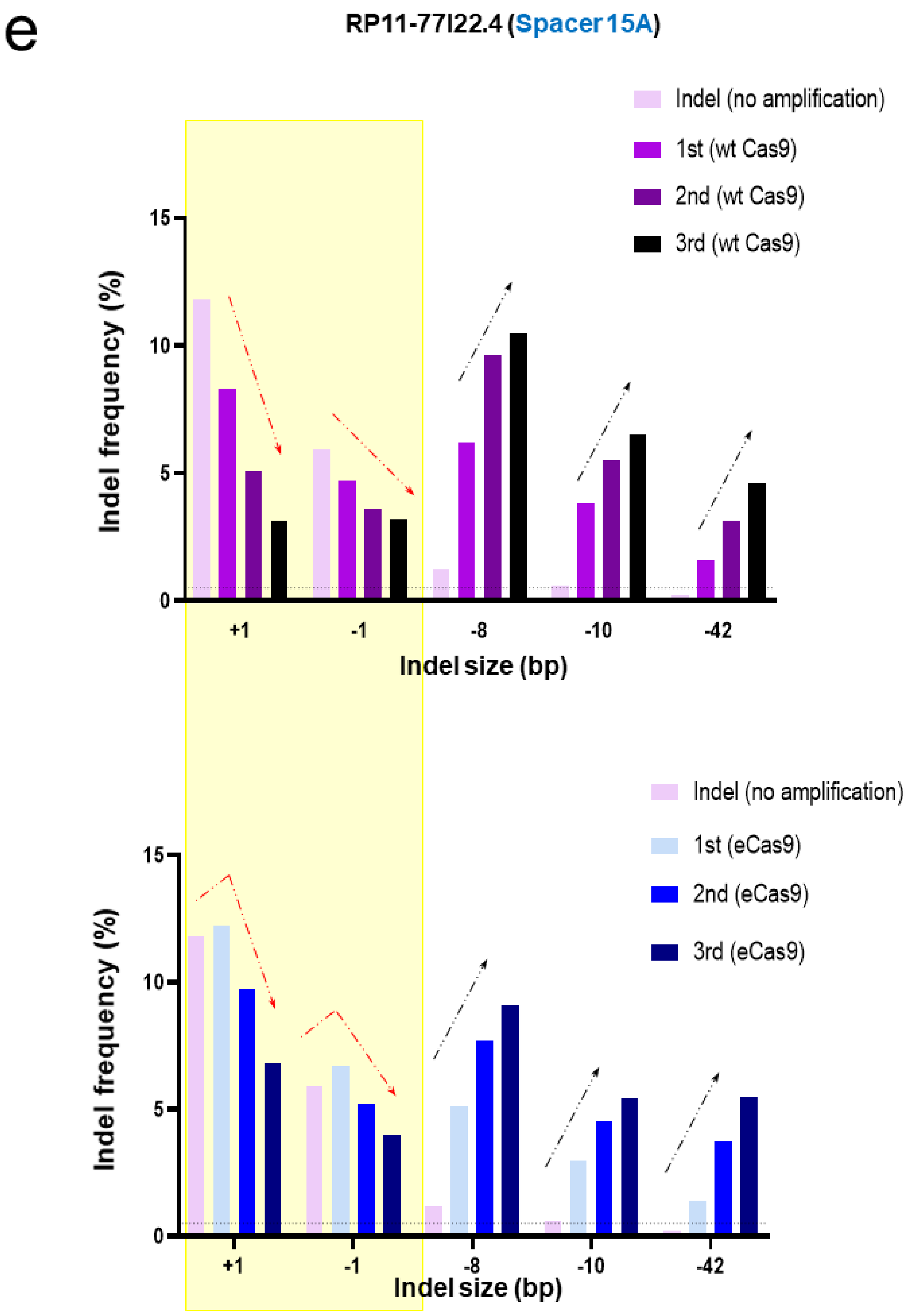

Supplementary Figure 11. Comparison of enrichment property between wild-type Cas9 (wtCas9) and specificity enhanced Cas9 (eCas9). (a) Mutant DNA

amplification with wild-type Cas9 (wtCas9) or specificity enhanced Cas9 (eCas9) for the target sequence (RP11-77122.4 site, Spacer15A) generated by the CRISPR-Cas9 effector in HEK293FT cells. Indel frequency (%) was analyzed by NGS after sequential amplifications with wild-type Cas9 (wtCas9) or specificity enhanced Cas9 (eCas9), respectively. Each amplification stage for mutant DNA enrichment is shown in light pink (no amplification, only indel), pink (1st CRISPR amplification), red (2nd CRISPR amplification) and dark red (3rd CRISPR amplification). Data are shown as mean from two (N=2) independent experiments. NC indicates negative control of no Cas9 delivery into cells. **(b)** Fold increases of mutations in RP11-77122.4 target for wild-type Cas9 (wtCas9) and specificity enhanced Cas9 (eCas9) after CRISPR amplification (N=2). **(c)** NGS analysis of CRISPR-Cas9 induced indel patterns on RP11-77122.4 (Spacer15A) locus. **(d)** NGS analysis of CRISPR-Cas9 induced indel frequency on RP11-77122.4 (Spacer15A) locus. Top: Indel frequency versus various size of indel patterns from NGS sequencing data (a). Middle: Each mutation frequency of amplification stage generated by wild-type Cas9 (wtCas9) is shown in pink (no amplification, indel only), light purple (1st CRISPR amplification), purple (2nd CRISPR amplification) and black (3rd CRISPR amplification). Bottom: Each mutation frequency of amplification stage generated by specificity enhanced Cas9 (eCas9) is shown in pink (no amplification, indel only), light blue (1st CRISPR amplification), blue (2nd CRISPR amplification) and dark blue (3rd CRISPR amplification). **(e)** A magnified histogram comparing the frequency of the mutation (from (d)) amplified using wtCas9 and eCas9, respectively. The yellow-highlighted portion shows the difference in tendency amplified by wtCas9 and eCas9.

Supplementary Fig.12

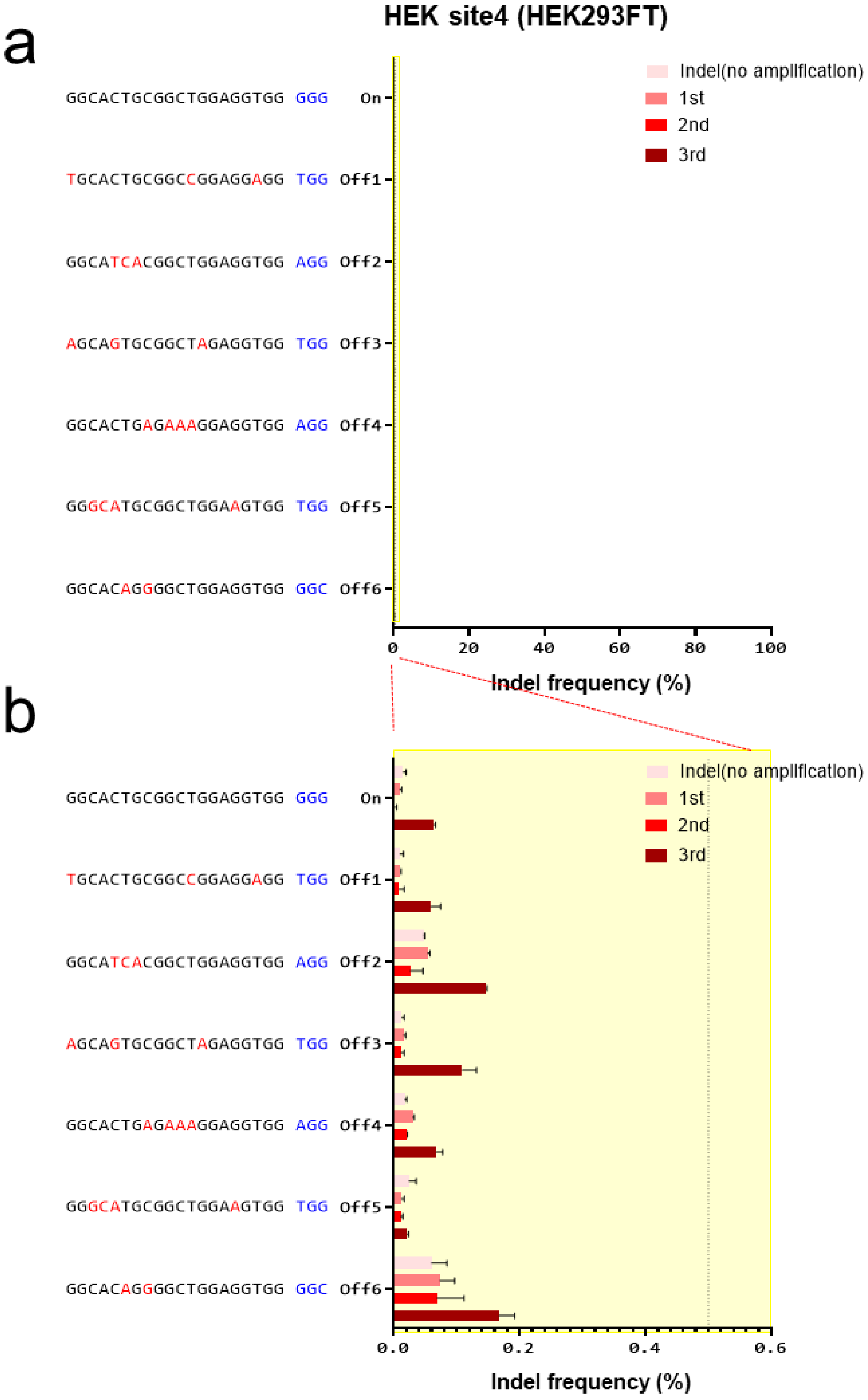

Supplementary Figure 12. The investigation of CRISPR amplification on negative control samples. (a) Detection of mutation frequency (%) for negative

control samples which is corresponding to the on/off target sequence (HEK293 site4) of Fig.3c. PCR amplicons were generated for on-target and six off-target sequences predicted *in silico* and the indel frequency (%) was analyzed by NGS after sequential CRISPR amplifications (N=2). NC indicates a negative control for no CRISPR-Cas9 delivery into the cells. **(b)** A magnified histogram of yellow highlighted region in (a). The dashed dashed line indicates the NGS detection limit (=0.5%).

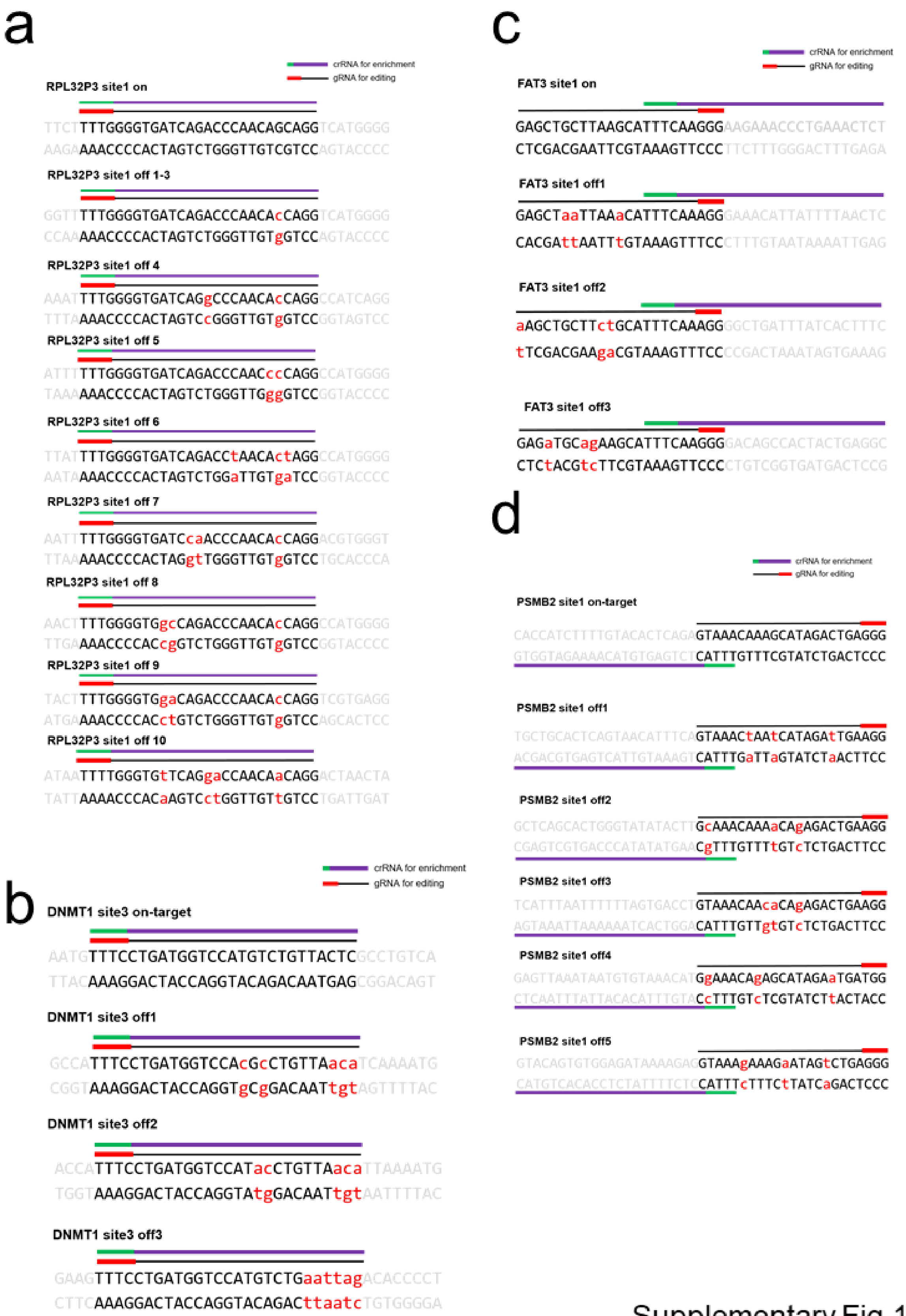

Supplementary Fig.13

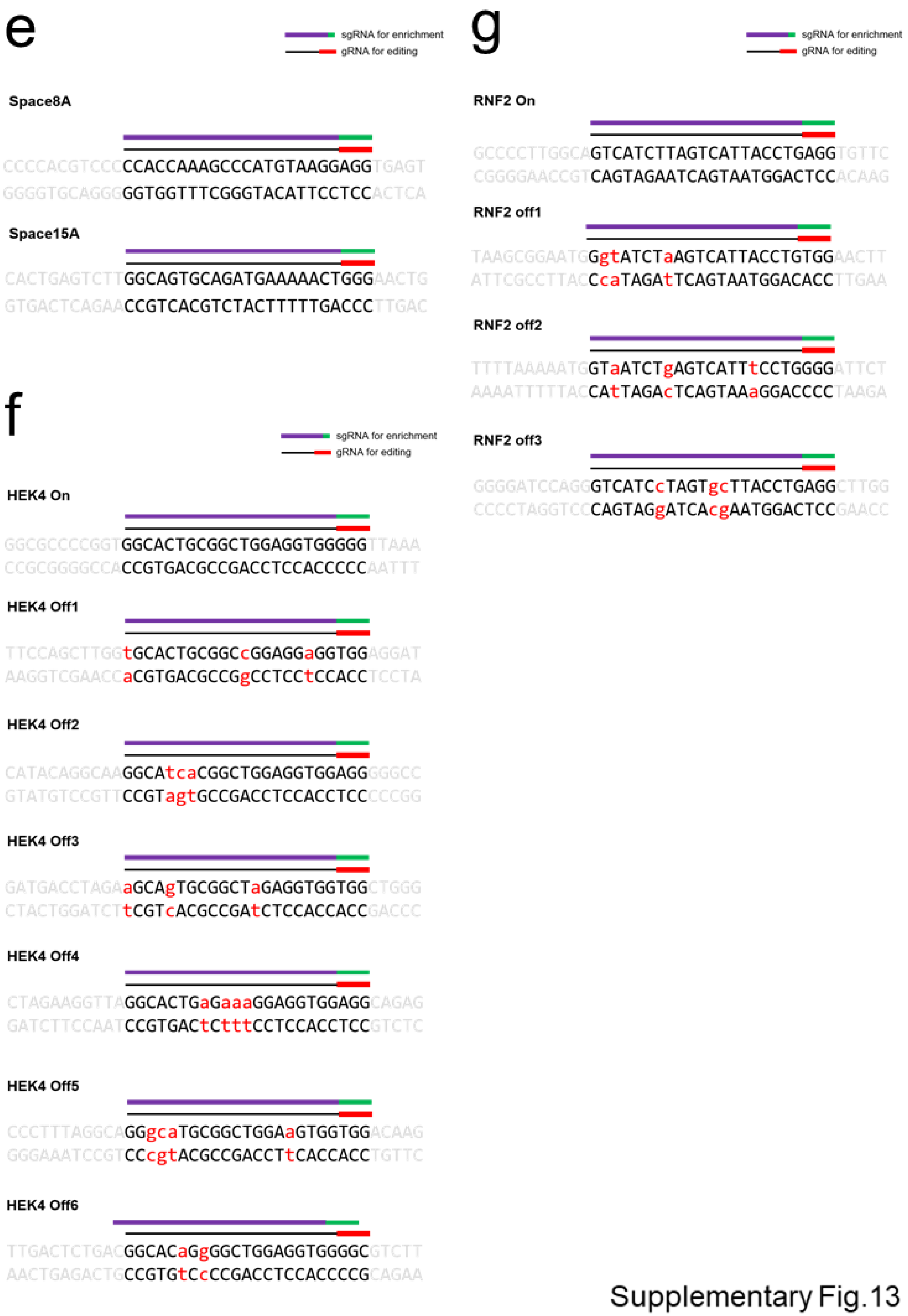

Supplementary Figure 13. Design of the guide RNA for wild-type DNA specific

cleavage and mutant DNA amplification. Guide RNA (gRNA) was used to induce target genomic locus mutation by various effectors and gRNA was used for target DNA enrichment by CRISPR amplification. Each single-guide and crRNA was designed for **(a)** *RPL32P3* on/off site mutations by Cas12a, **(b)** *DNMT1*-site3 on/off site mutations by Cas12a, **(c)** *FAT3* on/off site mutations by Cas9, **(d)** *PSMB2* on/off site mutations by adenine base editor, **(e)** Spacer8A, 15A site mutations by Cas9, **(f)** HEK293 site4 on/off mutations by Cas9, **(g)** *RNF2* on/off site mutations by Cas9. PAM sequence and mismatched sequences within protospacer region is shown in green, red bar and red lower case letter in protospacer, respectively.

References

1. Kleinstiver, B.P. et al. Genome-wide specificities of CRISPR-Cas Cpf1 nucleases in human cells. *Nat Biotechnol* **34**, 869-874 (2016).
2. Tsai, S.Q. et al. GUIDE-seq enables genome-wide profiling of off-target cleavage by CRISPR-Cas nucleases. *Nat Biotechnol* **33**, 187-197 (2015).
